# An adaptable neuromorphic model of orientation selectivity based on floating gate dynamics

**DOI:** 10.3389/fnins.2014.00054

**Published:** 2014-04-02

**Authors:** Priti Gupta, C. M. Markan

**Affiliations:** VLSI Design Technology Lab, Department of Physics and Computer Science, Dayalbagh Educational InstituteAgra, Uttar Pradesh, India

**Keywords:** feature maps, orientation selectivity, time-staggered WTA, floating gate synapses

## Abstract

The biggest challenge that the neuromorphic community faces today is to build systems that can be considered truly cognitive. Adaptation and self-organization are the two basic principles that underlie any cognitive function that the brain performs. If we can replicate this behavior in hardware, we move a step closer to our goal of having cognitive neuromorphic systems. Adaptive feature selectivity is a mechanism by which nature optimizes resources so as to have greater acuity for more abundant features. Developing neuromorphic feature maps can help design generic machines that can emulate this adaptive behavior. Most neuromorphic models that have attempted to build self-organizing systems, follow the approach of modeling abstract theoretical frameworks in hardware. While this is good from a modeling and analysis perspective, it may not lead to the most efficient hardware. On the other hand, exploiting hardware dynamics to build adaptive systems rather than forcing the hardware to behave like mathematical equations, seems to be a more robust methodology when it comes to developing actual hardware for real world applications. In this paper we use a novel time-staggered Winner Take All circuit, that exploits the adaptation dynamics of floating gate transistors, to model an adaptive cortical cell that demonstrates *Orientation Selectivity*, a well-known biological phenomenon observed in the visual cortex. The cell performs competitive learning, refining its weights in response to input patterns resembling different oriented bars, becoming selective to a particular oriented pattern. Different analysis performed on the cell such as orientation tuning, application of abnormal inputs, response to spatial frequency and periodic patterns reveal close similarity between our cell and its biological counterpart. Embedded in a RC grid, these cells interact diffusively exhibiting cluster formation, making way for adaptively building orientation selective maps in silicon.

## 1. Introduction

The past decade has been a landmark decade in the progress of Neuromorphic Engineering. Technological advances have paved the way for large scale neural chips having millions of neurons and synapses (Indiveri et al., 2006; Bartolozzi and Indiveri, 2007; Wijekoon and Dudek, 2008). We now have silicon cochleas and retinas (Chan et al., 2007; Lichtsteiner et al., 2008). A number of groups around the world have built large scale multichip neuromorphic systems for real time sensory processing with programmable network topologies and reusable AER infrastructure (Serrano-Gotarredona et al., 2005; Chicca et al., 2007; Merolla et al., 2007; Schemmel et al., 2008). All these approaches can be broadly classified into analog, digital or hybrid approaches. The analog approach interfaces well with the real world, emulates bio-inspired behavior more closely and is most suited for modeling local neural computations. Digital systems on the other hand efficiently exploit addressing mechanisms to emulate long distance communication in the brain. Therefore, an amalgamation of the digital and analog approaches i.e., the hybrid approach, is most appropriate for implementing large scale neuromorphic systems. The challenge that now lies ahead is to develop truly brain like cognitive systems. Systems that can adapt, self-organize and learn according to cues in their environment (Indiveri et al., 2009; Indiveri and Horiuchi, 2011).

A major step toward building such systems would be to understand the underlying principles that the brain uses to accomplish adaptation. It is well accepted now that very early in development the brain has a generic cortical structure that adapts to the environment by forming neural connections during the critical learning period (Sur and Leamey, 2001; Horng and Sur, 2006). This kind of adaptation leads to the formation of feature maps or interconnectivity patterns between hierarchically organized layers of the cortices. The lower layers extract basic features from the input space so that higher layers can extract more complex features, using the information from the lower layers. Both *Nature* (genetic biases) and *Nurture* (environmental factors) play a crucial role in the formation of these feature maps. Different hardware and software approaches have been explored to model self-organization. Each approach has a set of mechanisms that exploit the available techniques. While models built in software prefer to use mathematical equations, attempting to do the same in hardware can turn out to be extremely cumbersome (Kohonen, 1993, 2006; Martn-del-Bro and Blasco-Alberto, 1995; Hikawa et al., 2007). On the other hand, understanding the hardware dynamics and then building adaptive algorithms around it seems to be a more robust approach for building real world applications.

To emulate activity dependent adaptation of synaptic connections in electronic devices, we look towards the developing brain for inspiration. In the developing brain, different axons connecting to a post synaptic cell, compete for the maintenance of their synapses. This competition results in synapse refinement leading to the loss of some synapses or synapse elimination (Lichtman, 2009; Misgeld, 2011; Turney and Lichtman, 2012; Carrillo et al., 2013). Temporarily correlated activity prevents this competition whereas uncorrelated activity seems to enhance it (Wyatt and Balice-Gordon, 2003; Personius et al., 2007). Moreover, precise spike timing plays a key role in this process e.g., when activity at two synapses is separated by 20 ms or less, the activity is perceived as synchronous and the elimination is prevented (Favero et al., 2012). Apart from the biological relevance, synapse elimination as a means of honing neural connections is also suitable for implementation in large scale VLSI networks because in analog hardware it is difficult to create new connections but it is possible to stop using some connections. Although some digital approaches work around this by using virtual connections using the Address Event Representation, however, in purely analog designs for ease of management of large scale connections, synapse elimination is best suited. In order to implement synapse pruning we need to have non-volatile adaptable synapses which are best represented by floating gate synapse or memresistors (Zamarreño-Ramos et al., 2011). While memresistor technology is still in development floating gate transistors have gained widespread acceptance due to their capacity to retain charge for very long periods and the ease and accuracy with which they can be programmed during operation (Srinivasan et al., 2005). Floating gate memories are being used for various applications like pattern classification (Chakrabartty and Cauwenberghs, 2007), sensor data logging (Chenling and Chakrabartty, 2012), reducing mismatch (Shuo and Basu, 2011) etc. They have also found extensive application in neuromorphic systems (Diorio et al., 1996; Hsu et al., 2002; Markan et al., 2013). We therefore extend the study of adaptive behavior of floating gate pFETs and demonstrate how this adaptive, competitive and cooperative behavior can be used to design neuromorphic hardware that exhibits orientation selectivity, a widely studied phenomenon observed in the visual cortex.

**Figure 1:**
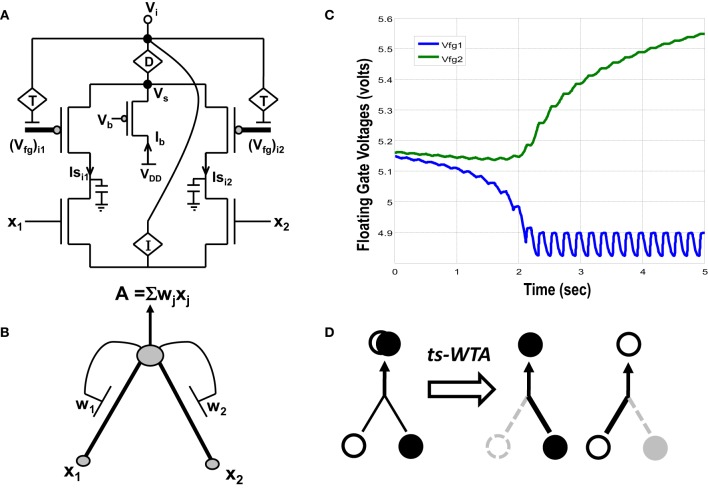
**(A)** Actual circuit of the ts-WTA learning cell and **(B)** its abstract model. In **(A)** (V_fg_)_i1_, (V_fg_)_i2_, and in **(B)** W1, W2 show the floating gate based weighted connections. x_1_, x_2_ are inputs and node voltage V_i_ is activation of the cell which is equivalent to A in **(B)**. **(C)** Shows ts-WTA evolution of floating gate voltages. **(D)** Starting with nearly equal weak connections (left), the cell strengthens stronger of the two connections at the cost of the other (right, shows both possibilities). Here ° => connection representing one feature • => connection representing other feature.

Prior efforts toward hardware realization of orientation selectivity can be classified into two categories, (1) Ice Cube models, (2) Plastic models. Ice cube models e.g., the model by Choi et al. (2005) assumes prewired feed-forward and lateral connections. Another similar model by Shi et al. (2006) uses DSP and FPGA chips to build a multichip modular architecture. They use Gabor filters to implement orientation selectivity. This approach provides an excellent platform for experimentation with feature maps, however, it falls short when it comes to compactness and power efficiency. Moreover, these models do not capture the developmental aspects of orientation selectivity. Some plastic models that try to capture the developmental aspects include the model by Chicca et al. (2007) that uses a mixed software/hardware approach to simulate a biologically realistic algorithm on a PC that is interfaced with a neuromorphic vision sensor. Another model by Boahen et al. (Taba and Boahen, 2002; Lam et al., 2005) uses activity dependent axon remodeling by using the concept of axonal growth cones and implements virtual connections by re-routing address events. Their design is biologically realistic but hardware intensive since they use an additional latency circuit to decide the wining growth cone. Therefore, what is needed is an approach that is more autonomous in terms of deciding the winner in the competition. Through our approach, that is based on the biologically inspired synapse elimination process, we have attempted to build an analog design that can be used by both analog and hybrid systems. The design has minimum hardware requirements and is capable of self-organized clustering. Our effort in designing a minimal competitive circuit, the time-staggered Winner Take All (ts-WTA) (Figures 1A–D) that exploits the adaptation dynamics of floating gate pFETs (Markan et al., 2013) and then using a collective network of these ts-WTA cells to exhibit orientation selectivity (Markan et al., 2007) is a small yet significant effort toward bridging the gap between biological phenomenon and its neuromorphic equivalent. The simulations were performed using Tanner T-Spice v13.0 and Cadence Specter v7.1 with BSIM3 level 49 spice models for 0.35 μm CMOS process.

Section 2 attempts to highlight the salient features of the ts-WTA circuit and discusses the motivation behind its design. Section 3 describes the development of a framework for multi-dimensional feature selectivity which is then extended to create an orientation selective cortical cell model that learns and eventually recognizes patterns resembling bars of different orientations. In sections 3 and 4, experiments performed on the orientation selective cortical cell, that highlight how close the cortical cell is to its biological counterpart, are discussed. Section 5 describes a framework for diffusive interaction and cluster formation between many orientation selective cells that has implications in orientation selective map formation. Section 6 includes the results and discussion.

## 2. Time-staggered winner take all

**Figure 2:**
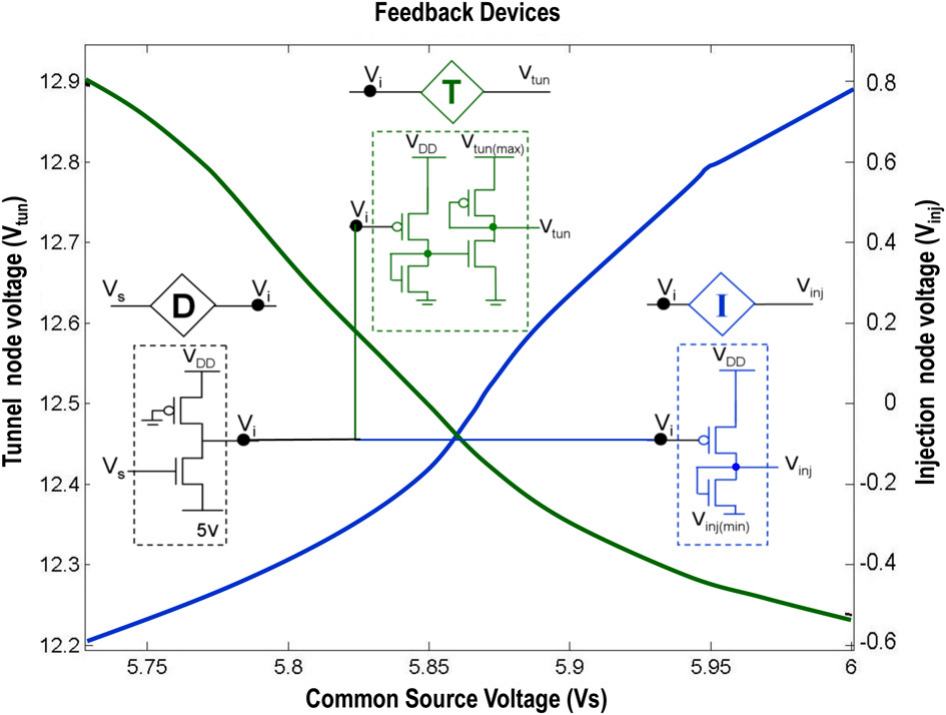
**Shows the circuit level description of the Injection (I), Tunnel (T) and Buffer (D) devices that are a part of the ts-WTA circuit shown in Figure 1A**. The Injection (I) and Tunnel (T) devices modify the voltage at V_i_ to appropriate ranges that enable injection and tunneling to occur in the floating gate pFETs. The buffer device (D) shields the common source node (V_s_) from the loading effect of neighboring cells. The graphs show how the tunnel (V_tun_) and injection (V_inj_) voltages vary with the common source voltage. Here V_inj(min)_ is set to −0.65 V, V_tun(max)_ is set to 13.6 V and V_DD_ is 6 v.

A novel CMOS time-staggered Winner Take All (ts-WTA) circuit has been described in Markan et al. (2013). The ts-WTA is built with two arms each representing a weighted connection, implemented by means of floating gate pFET “synapses” (Figure 1A) (Rahimi et al., 2002). These arms connect at a common source node, *V*_*s*_. Current through a bias pFET, also connected at *V*_*s*_, drives the two arms of the ts-WTA and ensures resource limitation. A buffer device (D) separating *V*_*s*_ from *V*_*i*_ is introduced to ensure that *V*_*s*_ is not influenced directly by the neighboring cells. However, the voltages at *V*_*s*_ and *V*_*i*_ are nearly the same. A feedback mechanism modifies the floating gate voltages of the two floating gate pFET synapses as a function of the activation node voltage *V*_*i*_. The Tunnel (T) and Injection (I) devices, that are a part of the feedback network (Figure 2), transform the *V*_*i*_ to appropriate ranges that make tunnel and injection feasible. The initial floating gate voltages of the two synapses are chosen randomly with a small voltage difference δ*V_fg_*. Inputs to the cell are applied in the form of pulses of high (6v) and low (−1v) voltage represented by 1 and 0, respectively. A {0,0} input means both the synapses are stimulated with −1v which is equivalent to saying they are both off. An input {1,1} means that both synapses are stimulated with a 6v pulse at the same time, which is how conventional WTA circuits receive inputs. The inputs {1,0} and {0,1} mean that the synapses are stimulated alternately or in an uncorrelated manner. The ts-WTA is designed to work on this uncorrelated scheme of inputs. When inputs from the sets {0,1} and {1, 0} are applied at *x*_1_ and *x*_2_ in a *random-inside-epoch* order (i.e., within an epoch both the synapses are equally stimulated but the order in which they are stimulated is randomized for every epoch) competition between the two arms starts taking place. The below equation expresses the adaptation dynamics of the floating gate voltage (*V*_*fg*_) of any branch (synapse) as a function of *V*_*fg*_ of the stimulated branch

(1) $$\frac{d{\left(V_{fg}\right)}_{ij}}{dt}=F_{T}\left[T\{V_{i}\},{\left(V_{fg}\right)}_{ij}\right]-F_{I}\left[I\{V_{i}\},{\left(V_{fg}\right)}_{ij}\right]\times x_{j}$$

The first part of the equation represents tunneling and the second part represents injection feedback. The second part has an additional term *x*_*j*_, which is 1 when the pFET is ON and 0 when it is OFF, taking into consideration that injection works only when the floating gate transistor is ON whereas, tunneling works at all times irrespective of the state of the floating gate transistor. In the first and second parts, *V_i_* is equivalent to $\sum_{j}f{\left(V_{fg}\right)}_{ij}\times x_{j}$ (which means we can express *V_i_* in terms of floating gate voltages of individual branches under the condition that only one *x_j_* is 1 the other is 0 at any given time). In the first part *T*{*V_i_*} leads to a tunnel voltage *V*_tun_ which along with the floating gate voltage (*V_fg_*) determines the tunneling current (*I*_tunnel_) and in the second part *I*{*V_i_*} leads to an injection voltage *V*_inj_ which along with *V_fg_* determines the injection current (*I*_injection_) (please refer to Markan et al., 2013 and Rahimi et al., 2002 for detailed equations). Injection works by lowering the floating gate voltage, *V_fg_*, thus making the transistor more and more ON whereas tunneling causes the *V_fg_* to increase gradually causing the pFET to slowly drift toward the OFF state. On stimulation by uncorrelated inputs over a period of time, injection amplifies the voltage difference between the two floating gates. Tunnel on the other hand helps in setting an upper limit on strength of the active connection, and pruning the strength of the inactive connection. According to Grossberg (1976), Winner Take All action requires that self-excitation of a neuron must be accompanied by global lateral inhibition. This occurs in ts-WTA with self-excitation in the form of injection and global lateral inhibition in the form of tunneling. If over many epochs, the synapse strengthens more than it weakens (there is more injection than tunneling), the floating gate pFET turns more and more ON, but if the synapse weakens more than it strengthens (tunneling is more than injection) then after several epochs it reaches a stage of no recovery where the floating gate pFET completely switches OFF. The synapse that strengthens more emerges as the Winner. However, ts-WTA has an additional interesting dimension according to which, if the weaker connection is stimulated more, then that emerges as the winner. Interestingly, this ts-WTA competition can be extended to any two contrasting input synapses (e.g., Left/Right eye in Ocular Dominance, ON/OFF cells in Orientation Selectivity and Lagged/Non-Lagged cells in Direction Selectivity) to perform feature selectivity. It can also be extended to other modalities like auditory, somatosensory etc. Thus the ts-WTA is a very generic cell and can be an essential core around which different feature selectivity models can be built. This is necessary for eventual integration of different feature maps into one universal framework. The ts-WTA has been studied under various stimulation schemes and has been tested for stability under device parameter variations (Markan et al., 2013) and is thus a robust circuit which closely emulates brain like competition and learning and is therefore suitable to build brain like feature maps.

**Figure 3:**
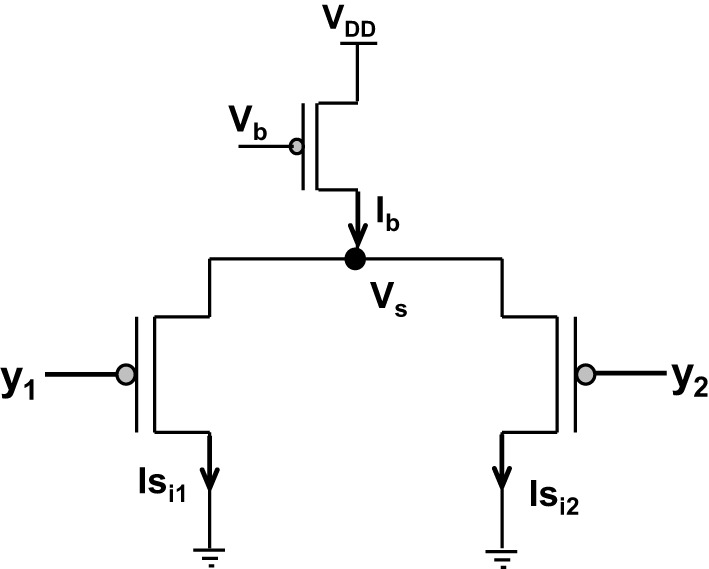
**Shows the Lazzaro's WTA (L-WTA) circuit**. This can be compared with the ts-WTA of Figure 1A. In both the circuits, a bias transistor in saturation acts like a current source with constant current I_b_. This current ensures resource limitation forcing only one of the input arms or “synapse” to survive in the competition. In ts-WTA the inputs x_1_ and x_2_ represent voltage pulses of +6 v and −1 v (1 and 0) applied alternately (time-staggered inputs) to both the arms. The inputs y_1_ and y_2_ are voltages (equivalent to x_1_w_1_ and x_2_w_2_, respectively) that are applied to the two arms of L-WTA both at the same time. Here w_1_ and w_2_ represent the weights of the two floating gate pFET synapses in the ts-WTA. L-WTA performs instantaneous comparison between the two inputs and does not have any memory element. The ts-WTA has floating gate pFET based memory and Tunnel and Injection feedback devices that modify the floating gate voltages as a function of response voltage (V_i_). This allows it to perform competition based on memory of prior activity unlike the L-WTA that can only perform instantaneous comparison between two inputs that are simultaneously applied.

Amongst the various CMOS WTA circuits that have been designed, Lazzaro's WTA(L-WTA) (Lazzaro et al., 1989) has gained widespread acceptance (Figure 3). It is an elegant circuit that performs instantaneous comparison between two or more input values and brings about suppression of the outputs associated with lower input values as compared to the highest value giving rise to Winner Take All action. Our ts-WTA is inspired by L-WTA, however, there are significant differences. In both the circuits, a current source restricts the amount of current that can flow in the two competing branches. As a result, the branch that draws more current forces the transistor of the other branch to switch off, thus emerging as a winner. When both inputs are applied at the same time, both ts-WTA and L-WTA behave in much the same way. However, ts-WTA brings in an interesting innovation in the form of long term memory retention using floating gate dynamics. So, in fact, the ts-WTA is a learning WTA cell that is capable of computing a winner based on which input is statistically more significant over many epochs unlike the L-WTA which only computes the winner based on an instantaneous comparison. Another interesting WTA circuit (inspired by L-WTA) that incorporates a sense of time by using floating gate transistors has been developed by Kruger et al. (1997). Their motivation to introduce adaptation is to add a fatigue or refraction time to each cell that wins. Their application is to form saliency maps where there is a need to ensure that the saliency of all inputs is considered and the WTA operation chooses different winners at different times instead of just locking on to the most significant input. Another interesting variant of L-WTA is the one introduced in Indiveri (2001, 2008). In this circuit by using local excitatory feedback and a lateral excitatory coupling mechanism the authors realize distributed hysteresis using which the network is able to lock onto an input with the strongest amplitude and track it as it shifts. They have shown an interesting application of this in adaptive visual tracking sensors (Indiveri et al., 2002). Both these circuits work on the conventional {1,1} or simultaneously applied inputs. They are both ingenious circuits, however, their motivation and design vary significantly from ours.

The true strength of the ts-WTA lies in the way it works on uncorrelated inputs or inputs applied staggered over time. The inspiration for using time-staggered or uncorrelated inputs comes from the way the brain is designed. In the brain, many pre-synaptic neurons connect to a single post-synaptic neuron through many afferent connections. It is only through correlated or uncorrelated activity between the many pre-synaptic cell afferents that the post-synaptic cell can tell to which pre-synaptic cell the afferents belong. The activity, from all the afferents of one pre-synaptic neuron is perfectly correlated whereas between two different pre-synaptic neurons the activities are uncorrelated and this is the basis on which synapse elimination happens (Stent, 1973). Hence, uncorrelated activity between different pre-synaptic neuron helps the post-synaptic neuron to decide, which connection is relevant and which is not. This selection process happens over a period of time and not instantaneously and therefore L-WTA is not suitable for such selection that involves retaining some information of prior neural activity. One of the most important aspect of neural information processing is feature extraction and formation of feature maps. Formation of feature maps requires that cells with similar feature selectivity cluster together. For this to happen each cell should be able to uniquely convey its feature preference at its output node which requires that each cell has to be identically stimulated by a selected pattern. Then on the basis of the responses of different cells for that pattern, cells that are selective to that pattern can be identified. Similarly, by applying other patterns cells can be marked for feature selectivity toward those patterns. Therefore, learning has to be deferred over an epoch so that all patterns are stimulated once and cells with similar feature preference can cluster together. In an L-WTA this is not possible for two reasons. Firstly, because in L-WTA inputs are applied simultaneously or in the {1,1} manner. This is analogous to applying all patterns at the same time and hence cells cannot be uniquely identified for their feature preference. Secondly, because the L-WTA lacks a mechanism for long term retention of modified weights which is needed for forming clusters. The ts-WTA on the other hand is perfectly suited as a learning cell for developing feature maps in silicon.

It may be apt to mention here that over and above facilitating synapse elimination, time-staggered or uncorrelated inputs play a major role in the formation of feature maps and this has been brought out in many seminal papers in neuroscience. For example Weliky and Katz (1997) reported that by artificially inducing correlated activity in both the eyes of the ferret, they found that the number of cells in the primary visual cortex with clear orientation and direction selectivity was markedly reduced when compared to un-stimulated controls. In a similar experiment on kittens, Stryker and Strickland (1984) found that segregation in ocular dominance columns was promoted when neural activity is synchronized in each eye but not correlated between the eyes. In other similar experiments on cortical feature map development in visual (Elliott and Shadbolt, 1998; Jegelka et al., 2006) as well as auditory cortex (Zhang et al., 2002) it has been reported time and again that spatiotemporal relation between the inputs to both eyes/ears are the key to formation of feature maps. Hence, it comes as a deduction from the above evidences that uncorrelated or “time-staggered” activity is an underlying biological mechanism for the formation of feature maps in the cortex. Therefore, using this inspiration to build artificial feature maps in silicon would help us bridge the gap between actual neural phenomenon and its neuromorphic equivalent.

The use of ts-WTA to build Ocular Dominance (OD) Maps has been described in Markan et al. (2013). In order to build a generic framework for cortical feature map formation in neuromorphic hardware, our ultimate goal, we wanted to extend our model to a larger input space. Orientation Selectivity (OR), a property exhibited by neurons in the visual cortex, is a natural extension to OD. OD is the selective preference cortical neurons show toward inputs from either the left eye or the right eye. The input space in OD is only two dimensional. OR on the other hand, is the selective preference cortical neurons show toward light or dark bars or edges of different orientations. Since orientations can vary anywhere from 0° to 180°, the input space is truly multi-dimensional. The following sections describe how from the basic building block of ts-WTA, we build an adaptable framework for multi-dimensional input features and how we extend it to build an adaptable circuit that is able to learn and eventually respond to different orientations.

## 3. Orientation selectivity

**Figure 4:**
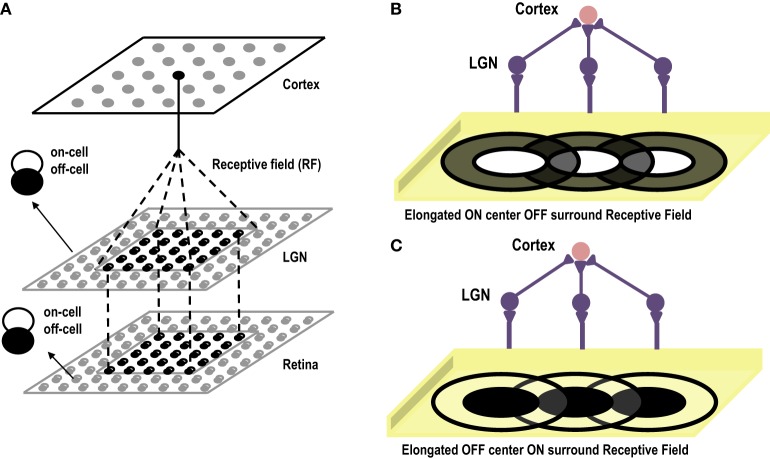
**(A)** Shows the three layer abstract feed-forward model of Orientation Selectivity. The first layer, retina, is the layer that receives inputs. The second layer is the LGN. There is one-to-one mapping between retina and LGN cells. The third layer is the cortex. Many LGN ON/OFF center cells innervate at a single cortical cell forming its receptive field. **(B)** Shows the elongated ON-Centered, OFF-Surround receptive field of a cortical cell (Inspired by Hubel and Wiesel's model of Orientation Selectivity). **(C)** Shows the elongated OFF-Centered, ON-Surround receptive field of a cortical cell.

Cells in the primary visual cortex are known to respond to dark and bright oriented bars. This property of the cortical cells, known as Orientation Selectivity, was first discovered by Hubel and Wiesel (1959). Hubel and Wiesel identified the receptive fields of Simple Cells in the Primary Visual Cortex and then showed bars of different orientations to the eye. Interestingly they observed that a single cell gave maximum response to a bar of only one particular orientation. They also observed that if the bar was in the center of the receptive field, it gave the highest response. In earlier experiments on retinal ganglion cells and lateral geniculate nucleus cells (Kuffler, 1953) it was observed that the receptive fields of these cells are divided into 2 parts (center/surround), one of which is excitatory or “ON,” the other inhibitory or “OFF.” For an ON/OFF center/surround cell, a spot of light shown on the inside (center) of the receptive field elicits spikes, while light falling on the outside ring (surround) suppresses firing below the baseline rate. Results are opposite for an OFF/ON cell. Hubel and Wiesel were proponents of the theory that receptive fields of cells at one level of the visual system are formed by inputs from cells at a lower level of the visual system, emphasizing that there is a hierarchical arrangement in the cortex, where in the higher layers extract statistically relevant information from the lower layers. Hence, they advanced the theory that small, simple receptive fields could be combined to form large, complex receptive fields. Later theorists also elaborated this simple, hierarchical arrangement by allowing cells at one level of the visual system to be influenced by feedback from higher levels. In their theory of orientation selectivity, Hubel and Wiesel proposed that Simple cells have receptive fields composed of elongated ON and OFF sub-regions (Hubel and Wiesel, 1959, 1962), which seem to originate from single synaptic input from ON and OFF centered lateral geniculate cells. The circularly symmetric receptive fields of neurons in LGN, that excite a cortical cell, are arranged in a row creating elongated receptive fields see Figures 4B,C. These elongated sub-fields are sufficient for generating a weakly tuned orientation response, which is then amplified by local intra-cortical connections. Unlike Ocular Dominance, that seems to develop only after eye opening, orientation selective responses have been observed to be present in primates, cats and ferrets as early as the first recordings can be made (Chapman et al., 1996). However, how the geniculate afferents organize themselves into segregated ON and OFF sub-regions during the prenatal period, in the absence of visual input, is still not clear. Some researchers attribute this development to spontaneous waves of activity that flow in the retina and LGN affecting cortical development (Mooney et al., 1996), and some attribute it to intra-cortical long range connections that exist before birth, forming a scaffold for orientation maps that later mature with visual inputs (Shouval et al., 2000). In order to gauge to what extent, visual experience influences the development of orientation maps, visual cortex of kittens reared in a single striped environment was studied using optical imaging techniques. It was found that even though kittens reared in a striped environment responded to all orientations, however, twice the area of the cortex was devoted to the experienced orientation as compared to the orthogonal one (Sengpiel et al., 1999). This effect is due to an instructive role of visual experience whereby some neurons shift their orientation preferences toward the experienced orientation. Thus, it is now generally accepted that although orientation maps are fairly stable at the time of birth, abnormal visual experience can alter the neuronal responses of a large percentage of cells to the exposed oriented contours. Under normal conditions, the prenatal tuning properties of neurons are retained and get refined with visual stimulus.

A number of models suggesting possible formation of orientation selective cells in cortex have been proposed. These have two main shortcomings. First, they employ a Mexican hat correlation function in the cortex (some use it in the LGN as well Miller, 1994). In the developing cortex, it is highly unlikely that this structure exists (Buzás et al., 2001; Yousef et al., 2001; Roerig and Chen, 2002). Second, competition in these models is brought in through synaptic normalization (multiplicative or subtractive). Normalization has its own associated problems, for linear synaptic weight update multiplicative normalization does not permit positively correlated afferent to segregate, while under subtractive normalization, a synapse either reaches the maximum allowed value or decays to zero (Miller and MacKay, 1994). These shortcomings have brought in the necessity of introducing models that are biologically more plausible (Miller, 1996; Elliott and Shadbolt, 1998). It has been observed that although the horizontal intra-cortical connections are still clustered at birth, the thalamo-cortical connections are well defined (Sur and Leamey, 2001). This indicates that the Orientation selectivity observed at birth could be manifesting out of the relatively well developed thalamo-cortical connections or the receptive fields of the cortical cell. These findings suggest the existence of some common biological mechanisms that could be responsible for the emergence of receptive field structure and thus orientation selectivity in the visual cortex. It has been shown that competition for neurotropic factors and neighborhood cooperation through diffusion of leaking chemicals (that lower the threshold of the neighboring cells and make them fire more readily on receiving same stimulus) are biological phenomenon acting in the brain both before birth and after (Cellerino and Maffei, 1996; Elliott and Shadbolt, 1998; McAllister et al., 1999). Models based on this competitive and cooperative behavior have been able to explain aspects of feature map formation of both orientation selectivity and ocular dominance (Markan, 1996; Bhaumik and Markan, 2000; Bhaumik and Mathur, 2003). Our model is inspired by the three layered model proposed by Bhaumik and Mathur (2003) (see Figure 4A for the abstract sketch of the model). However, there are some differences. While their model aims to describe the formation of oriented receptive fields prior to eye opening, our model also takes into account the influence of visual experience or cortical plasticity observed after eye opening. They use competition based on both pre and post synaptic resource limitation and diffusion between ON/ON center and OFF/OFF center cells, requiring precise initial connections between cells. Our resource limitation is only post synaptic and is enforced by limiting current in the bias transistor representing the cortical cell. The diffusion in our model happens between all neighboring cells irrespective of their type.

To build a hardware model of a cortical cell that exhibits orientation selectivity, from the building block of a single ts-WTA circuit, systematic scaling up was required. The next section describes how this scaling up was done and how diffusive interaction between ts-WTA cells was introduced.

### 3.1. Building a framework for multidimensional feature selectivity

**Figure 5:**
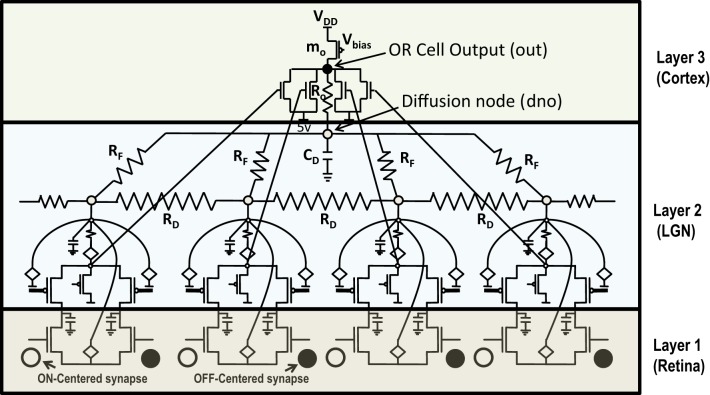
**Shows 4 ts-WTA cells connected in a row by means of diffusive resistors (R_D_)**. The output of each cell (V_s_) is connected in a feed forward manner using mosfets with their drains connected together at node ***out*** which is the feed forward path conveying the self activation or response of the cell. The activation node of each cell (V_i_) is connected at the diffusion node, ***dno***, with feedback resistances (R_F_). This forms the feedback network of the cell. A small resistance R_o_ connects ***out*** and ***dno*** to keep both these voltages nearly the same. The bias transistor m_o_ represents the cortical cell. Here V_DD_ is 6 v.

**Figure 6.1:**
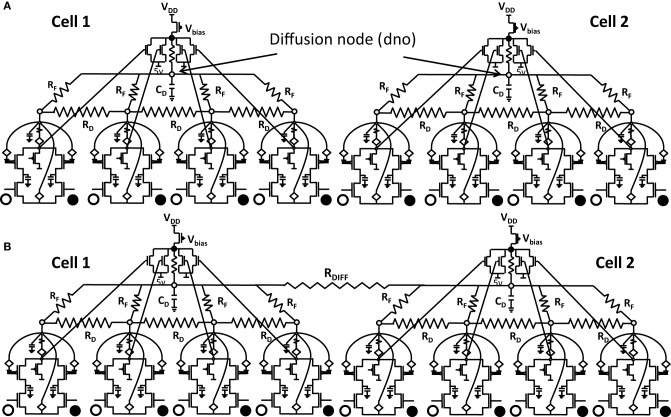
**Shows 2 cortical cells Cell 1 and Cell 2 with a 1 × 4 (ts-WTA) receptive field**. In **(A)** the two cells develop independently. In **(B)** the two cells are connected at the diffusion node (dno) by means of a resistance R_DIFF_ for diffusive interaction. Figures 6.2, 6.3 show how the receptive fields (floating gate voltages) evolve for the two cells in both the situations.

Any attempt at building self-organizing feature maps in hardware, requires neighborhood interaction to happen in such a way that local clusters are formed autonomously. We showed previously that this can be achieved by means of diffusive coupling between neighboring cells by means of an RC network (Markan et al., 2013). Biologically this happens through leaking chemicals from active neurons and as more recently shown through gap junction coupling (Li et al., 2012; Mrsic-Flogel and Bonhoeffer, 2012). In order to extend our design for feature selectivity over multi-dimensional input space, we took four ts-WTA cells and connected them in a row, with their outputs tied together in a feed-forward manner through MOSFETs (see Figure 5). This can be understood as a three-layered model where the first layer is the retina, the second layer is the Lateral Geniculate Nucleus (LGN) and the third layer is the visual cortex. While there is one-to-one mapping between cells in layer 1 and layer 2, there is many-to-one mapping from layer 2 to layer 3 cells, we call these layer 2 cells the receptive field of that layer 3 cortical cell. Therefore, now we have a cortical cell with a 1 × 4 receptive field. Individual ts-WTAs are connected to their neighbors with a 10k diffusive resistor (*R_D_*). The output of the cortical cell is fed back to the individual ts-WTA cells, through a resistive feedback network (*R_F_*), also of 10 k, as can be seen in Figure 5. The purpose of these resistances (*R_F_*) is to reinforce the initial bias so that the responses of the cells become fine-tuned ensuring that the pattern learnt is one of the applied patterns. The diffusion capacitor (*C_D_*) connected at node dno, is of 10 pF. To achieve cluster formation on a larger scale, it is important to achieve cluster formation locally. To ensure formation of local clusters within the set of four ts-WTA cells, the first and fourth ts-WTA of the cortical cell are connected diffusively in a ring fashion (not shown in the figure). This ensures that the receptive field develops into only one of the four patterns (0011), (1100), (0110), (1001), in which 11 and 00 are always clustered. We took 2 such cortical cells with a 1×4 (ts-WTAs) receptive field. The development of the receptive fields was analyzed in two situations. First, when the cortical cells are in isolation and second, when they are diffusively coupled with each other (Figures 6.1A,B).

**Figure 6.2:**
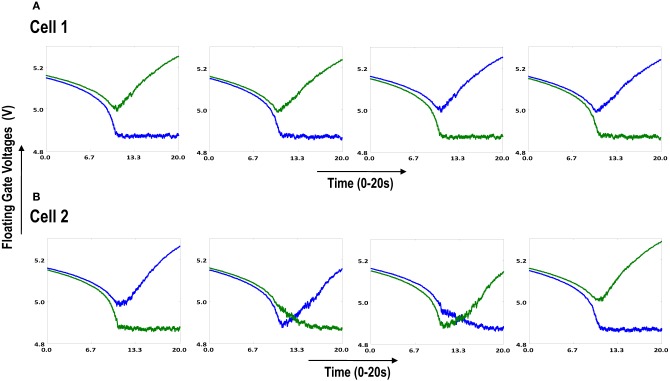
**Shows the development of floating gate voltages of the two cortical cells of Figure 6.1A**. Here blue represents the floating gate voltage of the ON-Centered synapse and green represents the floating gate voltage of the OFF-Centered synapse. The cells develop differently according to individual initial biases and inputs. **(A)** Shows the four ts-WTAs of Cell 1. The pattern of receptive field is 1100 and **(B)** Shows the four ts-WTAs of Cell 2. The receptive field has evolved into 0011.

**Figure 6.3:**
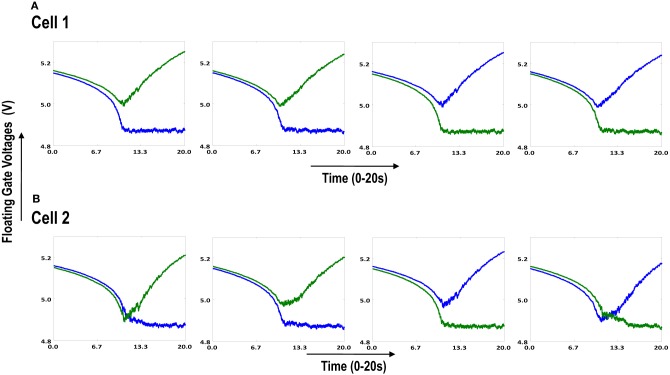
**Shows the development of floating gate voltages of the diffusively coupled cortical cells in Figure 6.1B**. Cell 1 which seems to have a stronger bias influences the development of Cell 2 which modifies its original response to become similar to cell 1. **(A)** Shows the unchanged response of cell 1 (1100) and **(B)** shows the response of cell 2 under strong influence of neighborhood (1100).

Both the cortical cells are stimulated with the same random-inside-epoch order of input patterns, however, their initial biases (initial floating gate voltages of LGN cells/layer 2) are different. The initial biases are randomly generated floating gate voltages varying between 5.15 and 5.16 v. We assume that the left branch of each ts-WTA represents an ON-Centered synapse and the right branch represents an OFF-Centered synapse. The inputs patterns are from the set (1100/0011), (1001/0110), (0110/1001), (0011/1100), (1010/0101), (0101/1010). The notation (1100/0011) means that when the ON-Centered synapses (left branches) of the four ts-WTAs of a cortical cell are stimulated by 1100 the OFF-Centered synapses (right branches) are stimulated by 0011 (as described in section 2, 1 here represents a high voltage (+6 v), and 0 represents a low voltage (−1 v) applied for 0.02 s). This is to emulate time-staggered or uncorrelated inputs. Please note that the patterns 0001 and 1000 are omitted from the set because they have unequal number of 0 s and 1 s and thus do not stimulate both the branches equally). When the input patterns are applied in a random-inside-epoch fashion, competition between the two arms of each ts-WTA cell begins. Depending on whether a branch is favored by the initial conditions more or is stimulated more or both, either the ON-Centered or the OFF-Centered branch wins. The resultant receptive field (i.e., the floating gate voltage profile of each branch of the four ts-TWAs) looks like one of the input patterns applied. Figure 6.2A represents the 1×4 receptive field of cortical cell 1 and Figure 6.2B represents the 1×4 receptive field of cortical cell 2 when they develop in isolation. When the two cortical cells are isolated, their receptive fields evolve into different patterns. Here cell 1' s receptive field has evolved into 1100 whereas cell 2' s receptive field has evolved into 0011. However, in the second case, when the two cells are diffusively coupled, their receptive fields evolve into similar patterns (1100) (Figures 6.3A,B). This happens because the diffusive node (dno) voltage, of the two cells becomes coupled. When the input patterns are applied, if one of the cells has a stronger bias for a particular input pattern the voltage at its node dno becomes high. Since both the cells receive the same random-inside-epoch order of inputs, the other cell also experiences this raised voltage at its node dno for the same pattern. The feedback resistors convey this high response back to the tunnel (T) and injection (I) devices (Figure 1A) which modify the floating gate voltages of all the ts-WTA cells reinforcing this pattern on them. Over many epochs, the difference between the ON-Centered and OFF-Centered branches of each ts-WTA cell gets amplified and the floating gate voltages get developed for the pattern that evoked the highest response at node dno during the initial few epochs. Towards which pattern the competition tilts occurs depends on the initial biases and the patterns applied and can be changed by changing either. Hence, promising results in the form of cooperation between neighboring cells are visible when two cortical cells are diffusively coupled.

**Figure 7:**
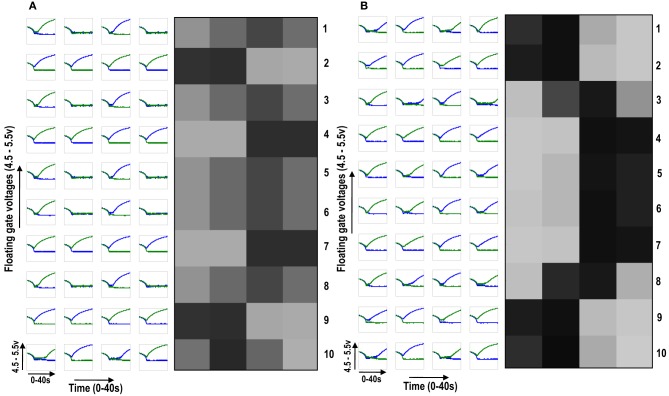
**(A)** Shows the development of floating gate voltages of 10 (1 × 4) ts-WTA cells in isolation. Here Cell 1 is top row, Cell 2 is 2nd row and so on. The black, white and gray squares represent the feature preference of the ts-WTAs. Black represents an OFF-Centered cell, white represents an ON-Centered cell and gray represents an unbiased cell. The cells develop differently according to individual initial biases and inputs. **(B)** Shows the same 10 cells when they interact diffusively. Near neighbor cells begin to cluster developing similar feature preference. Between two opposite patterns (e.g., 1100 and 0011), there is a gradual variation (1001), see responses of Cells 2, 3, and 4.

To see neighborhood cooperation and cluster formation on a larger scale we then diffusively connected 10 cortical cells, each with a 1×4 receptive field, with the tenth cortical cell connected to the first in a ring fashion. By giving all the cells different initial biases but subjecting them to the same sequence of random-inside-epoch patterns, interesting cluster formation was observed (see Figure 7). Figure 7A shows the development of the 10 cortical cells in isolation whereas Figure 7B shows their development under diffusive interaction. In the latter, two clusters of different patterns (or feature preference) are clearly visible. Between two opposite feature preferences (0011 and 1100), there is gradual variation between the feature preferences (1001) [see Figure 7B cells 2, 3, and 4 (rows 2, 3, and 4 from the top)]. With this idea of extendibility to multi-dimensional inputs and a framework for neighborhood interaction and clustering in place, we now build the circuit of a cortical cell that is capable of adapting and self-organizing, to become selective to patterns resembling different orientations.

### 3.2. Orientation selective cell model and simulation

**Figure 8:**
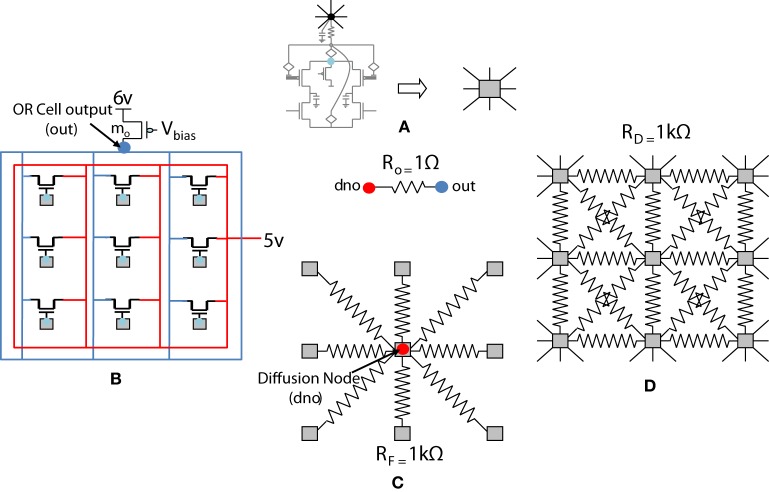
**Simplified and distributed layout of a 3 × 3 portion of the 9 × 9 receptive field of our orientation selective cell. (A)** Shows the symbolic representation of a ts-WTA cell. In subsequent figures, the gray square represents a ts-WTA. **(B)** Is the feed-forward MOSFET network that takes the output of the individual ts-WTAs and feeds them to the OR Cell output. This is a read out node from where self-activation of the cell can be recorded. **(C)** Shows the diffusive resistance network consisting of R_D_, which connects the ts-WTA cells to all their neighbors. **(D)** Shows the feedback resistive network consisting of R_F_ that feeds the output of the cell from dno back to the individual ts-WTAs. **Out** and **dno** are connected by R_o_ which can be replaced by a buffer device discussed in section 5.2. (see Figure 5 for the lateral view, this is a top view).

The previous section described the architecture of a cortical cell with a receptive field of 1×4 (ts-WTA) LGN cells. These cells when connected on an RC grid show diffusive interaction and cluster formation. The Orientation Cell model has a similar three layer topology, with retinal, LGN and cortical cells except that instead of a 1×4 receptive field, the orientation selective cortical cell has a two dimensional, 9×9 (ts-WTA), receptive field. However, with some differences in component values to balance out the effect of a larger neighborhood. The values of the diffusion, (*R_D_*), and feedback resistances, (*R_F_*), are now of 1k ohm each. A 3×3 simplified subsection of the circuit representing the receptive field of the cortical cell is shown in Figure 8. The capacitance connected at the node dno is 10 pF. The feed-forward MOSFETs connecting the common source nodes of the individual ts-WTA cells to the cortical cell (bias transistor *m*_*o*_) ensure that the self-activation of each cell is conveyed appropriately at the OR cell output, however, since there cannot be any current in the reverse direction, the OR cell's output will not affect the common source voltage at each ts-WTA. The purpose of the diffusive and feedback resistances remains the same i.e., to ensure proper neighborhood interaction and to fine tune the cell's response, respectively.

**Figure 9:**
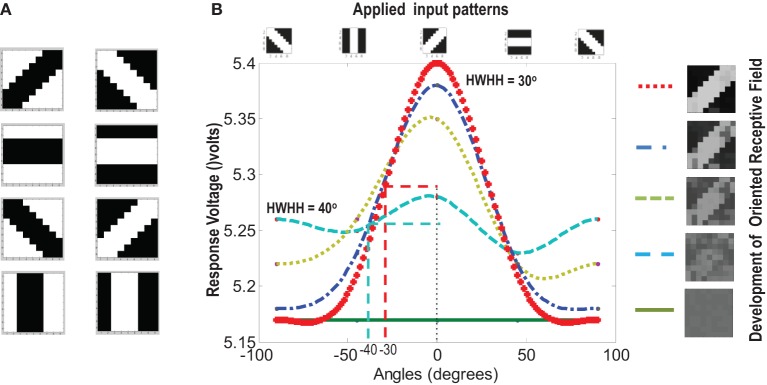
**(A)** Shows the input patterns that are applied to the orientation cell. **(B)** Shows the orientation tuning curve. Initially the response of the cell is low and similar for all input patterns. As the receptive field develops (see on the right, bottom to top), there is increased response toward that specific pattern as can be seen from the sharpening of the tuning curve. The half width at half height (HWHH) parameter for the best and the worst receptive field has been marked. The sharper the tuning, the lower is the value of HWHH.

A set of input patterns resembling ON-Centered and OFF-Centered oriented bars of angles 0°, 45°, 90°, and 135° were created (Figure 9A). Each pattern comprises of 9×9 blocks in which a bright block means stimulation with a +6 v pulse given for 0.02 s and dark block means stimulation by a −1 v pulse given for the same duration. To ensure that the learning is not biased towards the order in which patterns are applied, these bars were applied in a random-inside-epoch manner, however, with two constraints. (1) within each epoch when the left synapses of the 9×9 ts-WTA receptive field are stimulated with an ON-Centered oriented bar input, the right synapses are stimulated with the same orientation but with an OFF-Centered oriented bar. This is analogous to applying uncorrelated inputs to each ts-WTA branch and (2) just after that, this order is reversed, meaning, the left synapses are now stimulated with the OFF-Centered oriented bar and the right branches with the ON-Centered oriented bar of the same orientation angle. This is analogous to applying an orientation grating like input pattern that is necessary for orientation map formation. Gratings ensure that all the cells in the 9×9 receptive field are stimulated with the same oriented bar. This is necessary for cluster formation since clusters are formed when the cells group together according to similar feature preferences and whether two cells have the same feature preference or not can be known only when they receive the same inputs. Interestingly, the prenatal brain, when external inputs are absent, retinal waves have been identified to play the role of grating like input patterns that help in building a scaffold for orientation selectivity even before birth (Wong, 1999; Akerman et al., 2002). On the onset of simulation, the receptive field of the orientation selective cell i.e., 9×9 LGN cells are given random initial biases within 5.15–5.16 v. By applying the eight different input patterns in a random-inside-epoch manner, transient analysis on the circuit is performed for 80 epochs. As the simulation progresses, the synaptic connections from the ON-Centered and OFF-Centered LGN cells to the cortical cell compete and only one of the connections survives, the other gets eliminated (ts-WTA action). The local interaction between LGN cells is both competitive and cooperative. Competitive because of resource limitation in each ts-WTA cell, where only one of the connections (either ON-Centered or OFF-Centered) survives and cooperative by means of diffusive interaction between the neighboring ts-WTA cells, implemented by means of diffusive resistive coupling (*R_D_*) of the 9×9 ts-WTA cells, in a way similar to the Ocular Dominance model implementation. Details on the feedback mechanism acting on the floating gate pFETs in the individual ts-WTA cells and Ocular Dominance Map formation can be found in Markan et al. (2013). The orientation input pattern for which the voltage at node dno is the highest or a pattern that is statistically more significant gets reinforced through the feedback resistors (*R_F_*) and the injection and tunnel feedback mechanisms of each ts-WTA cell (as discussed in the case for a 1×4 receptive field) and we say that the cell is selective to that particular orientation. Multiple simulations performed with different random initial biases of LGN cells (floating gate voltages) and different random-inside-epoch order of input patterns result into the cell learning different oriented patterns with equal likelihood of learning any one of the applied eight patterns. A statistical analysis over 100 simulations is presented in Tables 1A, 1B. The results show that each of the eight patterns is learnt at least 10% of the times. A video of how the receptive field of the orientation cell evolves, starting from initial random biases of LGN cells to an oriented bar pattern, can be found in the supplementary material.

**Table 1A:**
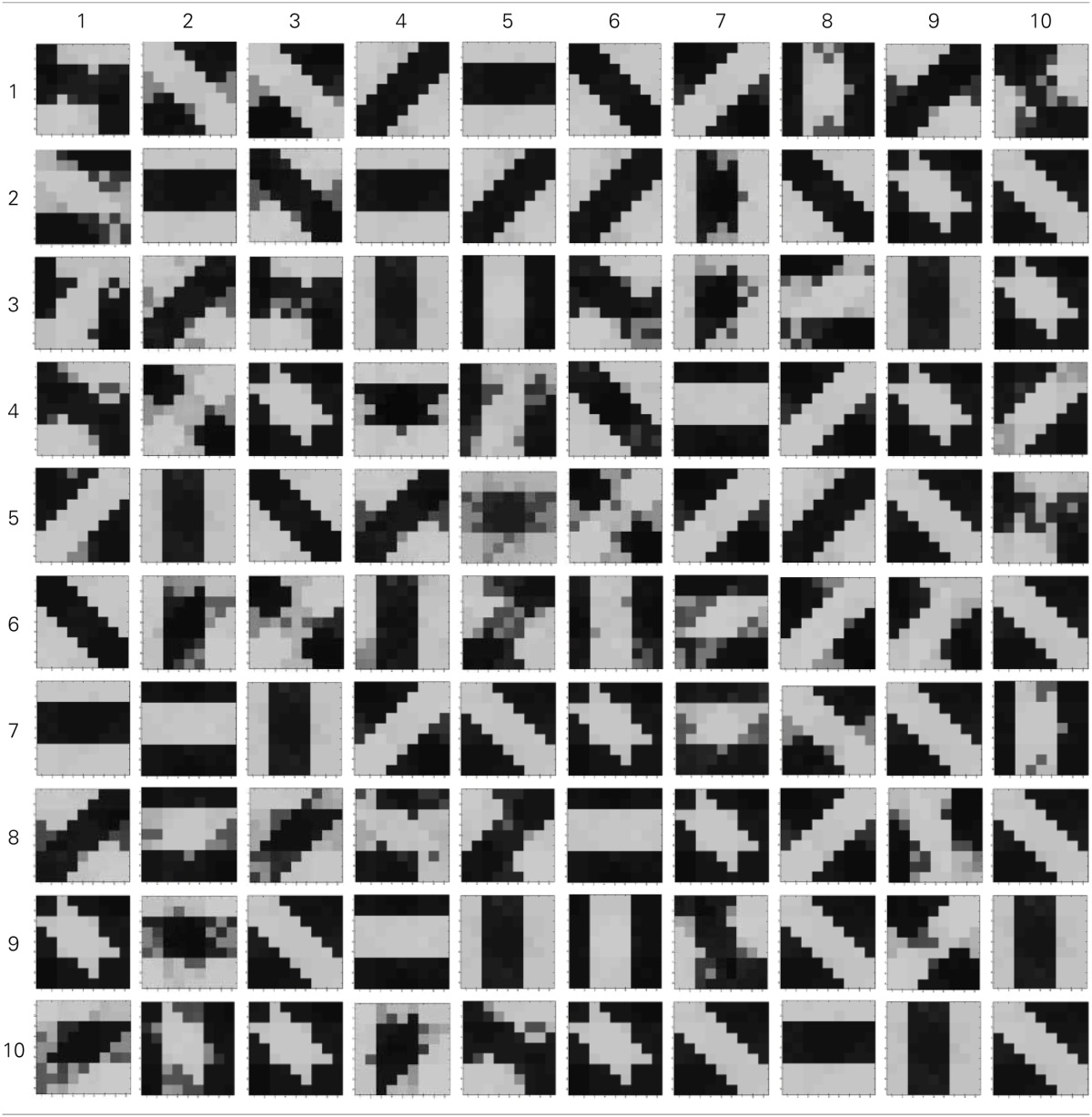
**Results of 100 simulations of the orientation selective cell performed with different random initial biases and different random-inside-epoch inputs**.

**Table 1B:**
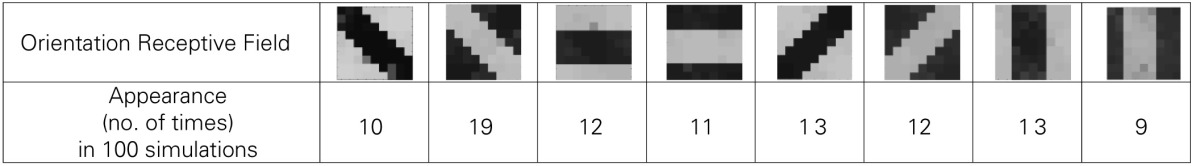
**Analysis of 100 simulations**.

^*^The evolved receptive field sometimes resembles two different orientations. In such cases the response towards both the orientations was noted and HWHH was computed in each case. The categorization was done on the basis of the lower HWHH.

### 3.3. Orientation tuning and performance under abnormal stimulation

Experiments done on many mammals demonstrate that during the early postnatal periods, the recording over a cortical neuron shows nearly equal response to many orientations or only slight bias toward a particular orientation. If the response of the cell is plotted against different orientation angles, it is a flat curve showing faint selectivity to many different orientations. As the orientation selectivity of the cell develops, as a result of stimulus dependent activity, the tuning curve becomes sharper at a particular orientation (Somers et al., 1995; Dragoi et al., 2000; Seriès et al., 2004). Similar orientation tuning is exhibited by our orientation selective cell. Once the cell has learnt a particular orientation i.e., the floating gate voltages of the cell have matured, the injection and tunnel voltages can be modified in a way that stops further learning, see learning rate parameter in Markan et al. (2013). The cell's response to any orientation can then be obtained by observing the output node voltage (OR cell output node) on the application of that oriented pattern as input. Figure 9B shows the orientation tuning curve of our cell at different stages of receptive field development. The development of orientation tuning is clearly visible from the shape of the curve. Initially the cell responds equally to all orientations, depicted by the nearly flat curve, gradually becoming selective to only one, represented by the rising peak at one of the orientations. The *Half Width at Half Height* (HWHH) was computed for each receptive field for the 100 simulations mentioned in the previous subsection. For the receptive fields that were not very finely tuned or they seemed to be close to more than 1 input patterns e.g., receptive field (5,5) in Table 1A, the HWHH was computed for each case and the receptive fields were categorized (see Table 1B) according to the lower HWHH value. The best HWHH, i.e., the HWHH for a highly tuned receptive field e.g., (1,4) in Table 1A is 30° and worst HWHH is 40° for a receptive field similar to (5,5).

Some experimental results also suggest that if on the onset of vision, animals are reared in an abnormal environment such as one with only single stripes, the orientation tuning of a large number of cells, that were initially tuned to different orientations, adjust their tuning to respond to the orientation of the striped environment in which they are reared (Sengpiel et al., 1999; Yoshida et al., 2012) and the cortical space that was initially shared equally by all orientations now becomes exceedingly large for the orientation shown. In other words the orientations shown take up the cortical space of the orientations that were never shown. To test if similar behavior is shown by our orientation selective cell, two sets of experiments were performed. In the first experiment, for 20 different initial conditions, 8 different orientation patterns were applied. It was found that for 20 simulations, the receptive fields developed into one of the eight patterns, with nearly equal probability. Now, for the same set of initial conditions, we applied only six patterns (two horizontal patterns, 1 ON-Centered and one OFF-Centered were omitted). The results are summarized in Tables 2, 3. It was observed that for 20 simulations, the cell now developed according to the 6 patterns applied with nearly equal probability. Therefore the space that was earlier occupied by eight patterns was now equally distributed amongst six patterns. The cell demonstrated adaptive cortical plasticity by developing receptive fields according to the applied patterns. However, if the initial biases very strongly favor one of the missing patterns, like in Table 3, 2nd row 4th column, the receptive field develops according to the initial bias rather than the applied patterns. This kind of adaptive plasticity to accommodate abnormal inputs may not be possible in the model by Bhaumik and Mathur (2003) since their model does not take into account the effect of external stimulation.

**Table 2:**
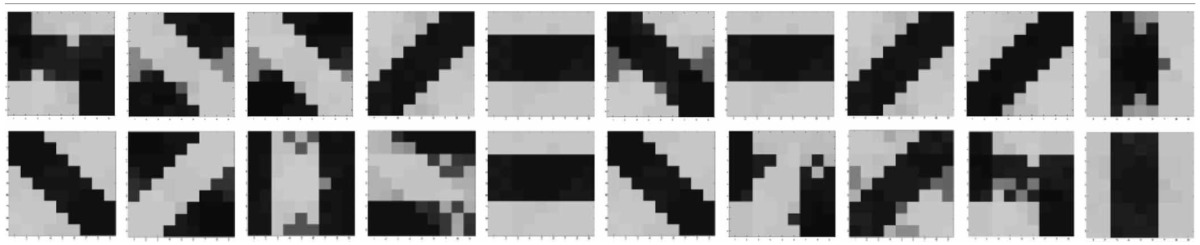
**Summary of 20 simulations of orientation selective cell with all 8 oriented patterns applied as inputs**.

**Table 3:**
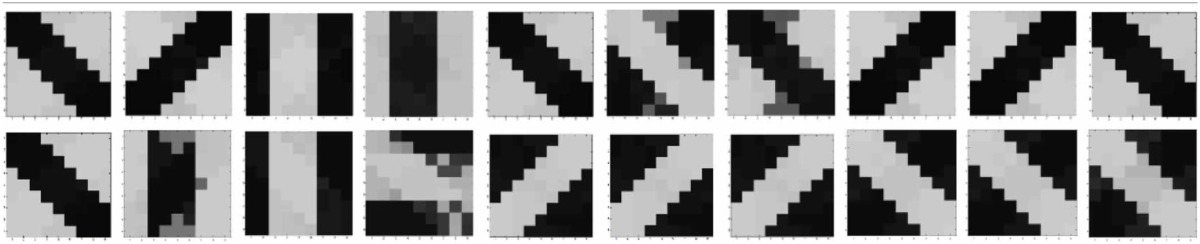
**Summary of 20 simulations of orientation selective cell with horizontal patterns missing**.

### 3.4. Analyzing the effect of nature *v_s_* nurture

It is known that both Nature (genetic biases) and Nurture (environmental factors) play an important role in feature map formation. To understand how our orientation selective cell responds to nature (initial biases) vs nurture (pattern stimulation) and to gauge how close it is to biology, two sets of experiments were performed. In the first experiment, repeated simulations were performed by keeping the initial biases over the 9×9 LGN cells the same, but changing the random-inside-epoch order of input patterns over all the epochs. Statistical analysis over 20 simulations showed that 80% of the times the cell learnt a different oriented pattern, highlighting that stimulus driven activity can override the orientation bent due to the initial floating gate voltages in most of the cells. In the second experiment, the random-inside-epoch order in which inputs are applied was kept constant (creating preference for one of the patterns) over all the simulations but the initial biases were changed every time. It was observed that although 70% of the times the cell developed the same oriented receptive field, but 30% of the times it did learn other patterns. This experiment brings out that it is not just the input patterns applied, but the unique combination of the inputs and the initial biases that decides which oriented pattern the cell would learn or become selective to, bearing close analogy to experimental findings. The results are summarized in Tables 4A, 4B.

**Table 4A:**
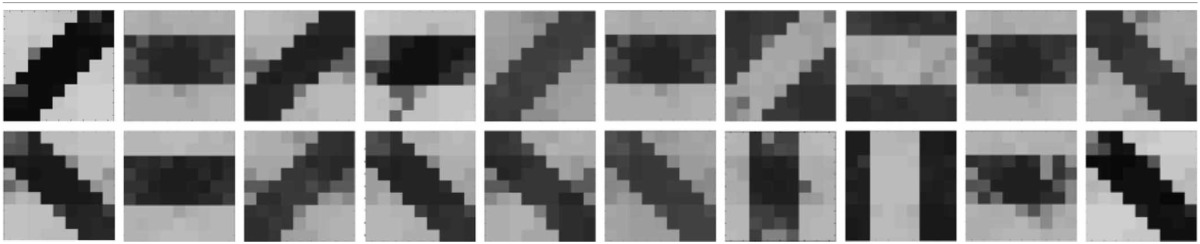
**Summary of 20 simulations of orientation selective cell with same initial conditions but different random-inside-epoch order of input patterns**.

**Table 4B:**
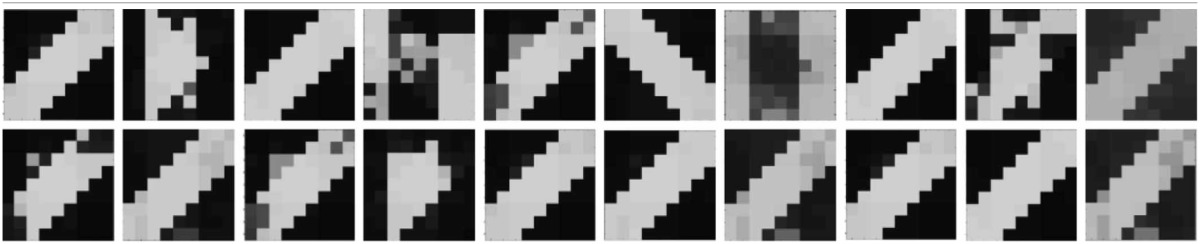
**Summary of 20 simulations of orientation selective cell with different initial conditions but same random-inside-epoch order of input patterns**.

## 4. Response to spatial frequency and periodic patterns

**Figure 10:**
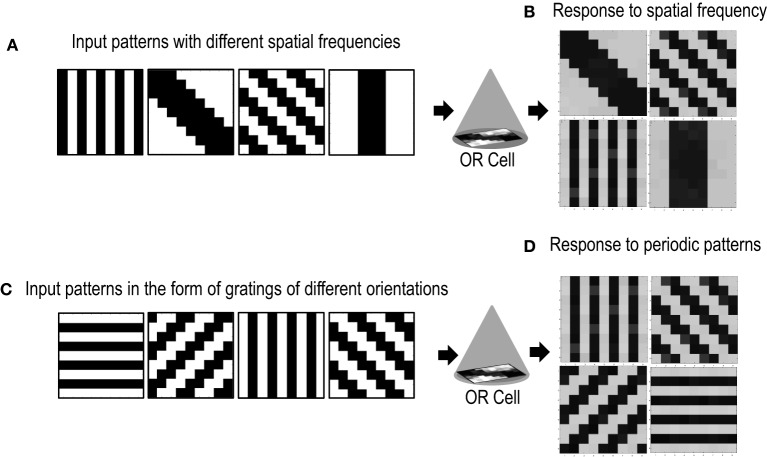
**Shows the response of the Orientation Cell to patterns of different spatial frequencies and periodic patterns of different orientations. (A)** Shows patterns of different spatial frequencies that are applied as inputs to the OR Cell. **(B)** Shows the patterns that the cell learns. Each simulation results in the circuit learning one of the input patterns with equal probability. **(C)** Shows periodic patterns of different orientations that are applied as inputs to the OR cell. **(D)** Shows the periodic patterns that the cell learns.

Cells in the primary visual cortex are also known to respond to the spatial frequency of visual inputs (Maffei and Fiorentini, 1973; Tootell et al., 1981; De Valois et al., 1982; Everson et al., 1998). Some cells respond to low spatial frequencies, some to high spatial frequencies, essentially forming spatial low pass, band pass and high pass filters that act on the visual inputs. To test if our cell could also be selective to the spatial frequency of applied inputs, we presented the circuit with patterns of different spatial frequencies (Figure 10A). The simulations were performed in the same way as described in section 3.2 except for the new input patterns that have orientations of different spatial frequencies. We took only two spatial frequencies (low and high). Repeated simulations resulted in the cell learning orientations of different spatial frequencies (Figure 10B). However, it was observed that the learning time of the cell increased as compared to when all inputs are of the same spatial frequency.

Certain cells in the visual cortex are also known to be selective to periodic patterns (Von der Heydt et al., 1992). These cells respond vigorously to gratings but not so much to bars or edges. Since these cells are not sensitive to the spatial frequencies of the gratings but are only specialized for detection of periodic patterns, they seem to have a role in the perception of texture. In order to test if our circuit could have a similar response to periodic patterns, we presented our circuit with input patterns that resembled gratings of different orientations (see Figure 10C). After several epochs of it was observed that the cell's receptive field developed according to one of the grating patterns (Figure 10D). Repeated simulations with different initial biases and different random-inside-epoch order of inputs resulted in the cell's receptive field evolving into one of the eight grating patterns with equal probability. These experiments show that the cell developed is generic and is extendable to recognizing many different patterns.

## 5. Diffusive interaction of cells

Feature map formation is based on three important tenets: *continuity*, *diversity* and *global order*. Continuity requires that nearby cells share the same feature preference. Diversity means that there is equal representation of all possible feature preferences and global order implies that there is a periodic organization of different features over the entire cortical surface. Literature sites several mechanisms that coordinate the development of feature selectivity of single cells under neighborhood influence (Grossberg and Olson, 1994). The essence of these mechanisms is that if cells have overlapping receptive fields and they receive similar inputs, then if they can be forced to have similar responses, Hebbian Learning mechanism will ensure that the individual cells' receptive fields develop to form clusters. As discussed earlier, this poses certain requirements on the behavior of the learning cell and the neighborhood function. Firstly, it demands that the learning cell should allow modulation of its feature selectivity under neighborhood influence. Secondly, it demands for a neighborhood function that is capable of generating an appropriate signal that can modulate the development of feature selectivity of a cell in concordance with other cells in the cluster.

Diffusive-Hebbian learning based on the biological phenomenon of reaction-diffusion has been shown to be effective in forming clusters of cells with similar feature preference and has also been used to model Ocular Dominance and Orientation Selectivity Map Formation (Markan, 1996; Krekelberg, 1997; Markan and Bhaumik, 1999; Bhaumik and Markan, 2000; Bhaumik and Mathur, 2003). Biologically, this happens by means of leaking chemicals coming out of an active cell, that lower the threshold of the neighboring cells. Reaction-diffusion can be easily implemented by an RC network as shown in Shi (2009) and Markan et al. (2013). The development of individual cells and cells under diffusive interaction varies significantly. If the cells have different initial biases then in the absence of diffusive coupling they develop into cells with different orientation preferences. On the other hand, the presence of diffusive coupling causes nearby cells to have a similar voltage (at node dno) and hence the injection and tunnel feedback that they receive is also the same. Therefore, if the two cells receive similar inputs, they develop to have similar feature preference. The stronger cell (the cell that generates a higher voltage at node dno) tends to influence the development of the weaker cells around it.

### 5.1. Modification of orientation tuning under neighborhood influence

As discussed earlier, for any map formation, diffusive interaction between cells should happen in such a way that it leads to formation of clusters of cells having similar feature selectivity. This is possible if some of the cells change their feature preference when they are surrounded by strongly biased cells, forming clusters showing gradual variation in orientation selectivity between clusters. This means that some kind of mechanism needs to be present that helps the cell in overcoming its initial orientation bias to develop an orientation preference according to the neighborhood influence. In our orientation cell this is achieved by ensuring two things. (1). Keeping the time constant of the diffusive RC network (τ_Diffusion_) much smaller than the time constant of the orientation cell (τ_Reaction_) and (2). Limiting the amount of learning in each iteration by applying input patterns for a very short duration (0.02 ms). The first condition ensures that diffusion has precedence over reaction and the strong neighborhood influence is able to modify the individual bias of an orientation cell and the second condition makes sure that the learning in the orientation cell is at a pace that is suitable for diffusion to influence its development i.e., the floating gate voltages are allowed to change by only a small amount in every iteration. This is required because once the difference between the floating gate voltages of the two arms of the ts-WTA becomes large, it cannot be reversed.

It may be noted that the diffusion node (dno) voltage varies between 5.1 and 5.4 volts as the receptive field develops. After development, the response of a developed cell to the pattern that it favors, measured at the diffusion node (dno) is around 5.4 volts. Interestingly, if we apply 5.4 volts to node dno externally, for a pattern of our choice, and do this repeatedly, the circuit begins to develop preference for that orientation instead of its natural bias. Therefore, the receptive field development of the orientation selective cell can be modulated externally by applying appropriate voltage at the dno node of the cell for a particular pattern. This way we force a high response for a pattern of our choice, which causes the feedback mechanism to reinforce the desired pattern on to the individual ts-WTA cells in the 9×9 receptive field. It was observed that as the floating gate voltages become more developed (developed floating gate voltages mean that the difference between the floating gate voltages of the two synapses of the individual ts-WTA cells has become large) it becomes difficult to modulate the orientation preference of the cell. For fully developed floating gate voltages, i.e., strong orientation preference, modulation does not happen at all, and the cells preserve their original response as expected. Details of how the floating gate voltages vary during unlearning and the influence of injection and tunnel voltages are examined critically in Markan et al. (2013).

### 5.2. Buffer device for diffusive coupling

**Figure 11:**
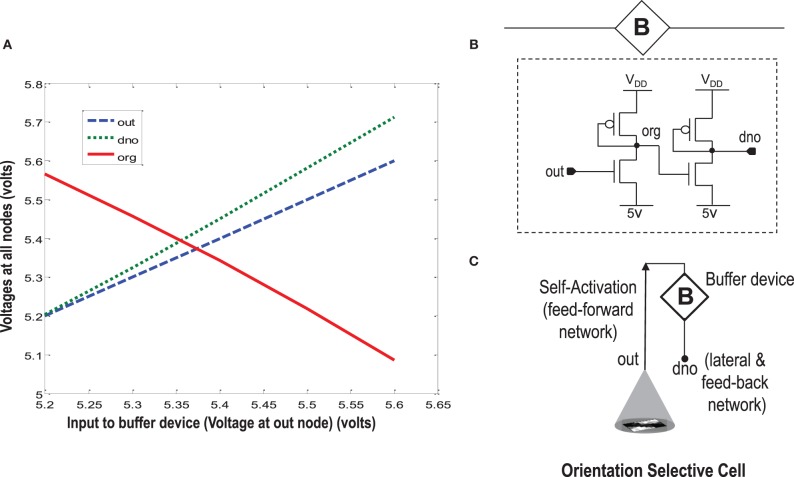
**In order to isolate the OR cell output (out) which conveys the self-activation of the cell, from the diffusion node (dno) at which other orientation cells connect and to prevent loading of node out, a buffer device is created. (A)** Shows the characteristic response of the buffer device. The device is linear, and has a double inverting effect on the voltage at node *out*. The V_DD_ is 6 v. **(B)** Shows a typical design of the buffer device. **(C)** Shows an abstract symbol for orientation selective cell along with the buffer device.

When more than one orientation cells are connected with each other diffusively using resistances at the diffusion node (dno), the increased current at the node dno tends to undesirably load the output node or OR cell output (out) (Figures 5, 8). Since the OR cell output (out) node conveys the self-activation of each cell, this value should not get altered. In order to avoid this loading effect, we designed a buffer device (B) that shields the orientation cell output (activation) from the excessive current coming to the node dno of each orientation cell from other diffusively coupled cells. This device ensures that the self-activation (feedforward network) of the orientation cell driving the voltage at node out can influence the voltage at node dno, that drives the feedback network, but node dno cannot influence the voltage at node out directly. This buffer device is essentially a linear device that inverts the voltage at the OR cell output (out) twice and feeds it to the dno node (see Figures 8, 11). This way current only flows in one direction, i.e., out of OR cell output node and not into it. A typical design of the buffer device is shown in Figure 11B, however, any other device performing the same function can be used as well.

### 5.3. Simulation of diffusive interaction between cells

**Figure 12:**
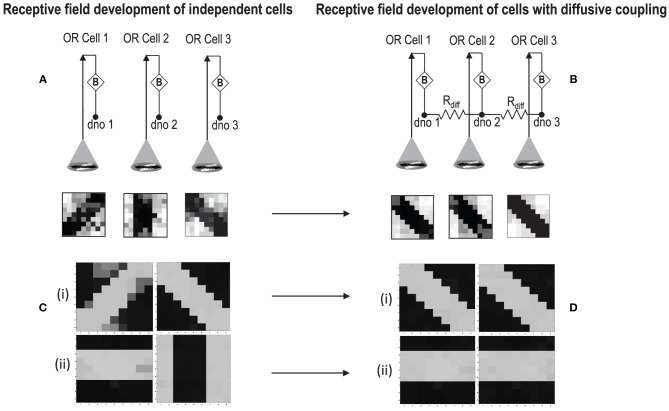
**(A)** Shows the independent development of receptive fields of three orientation selective cells with different initial biases and same random inside epoch order of inputs. **(B)** Shows the development of the same three cells with the initial conditions and order of inputs same as **(A)**, but with diffusive interaction between neighbors. All the cells develop similar feature preference. **(C)** Two more example of cells developing independently under the same random inside epoch order of inputs but different initial biases. **(D)** Shows the development of the same cells as **(C)** under diffusive coupling. Diffusion causes the cells to develop the same feature preference in each case.

**Figure 13:**
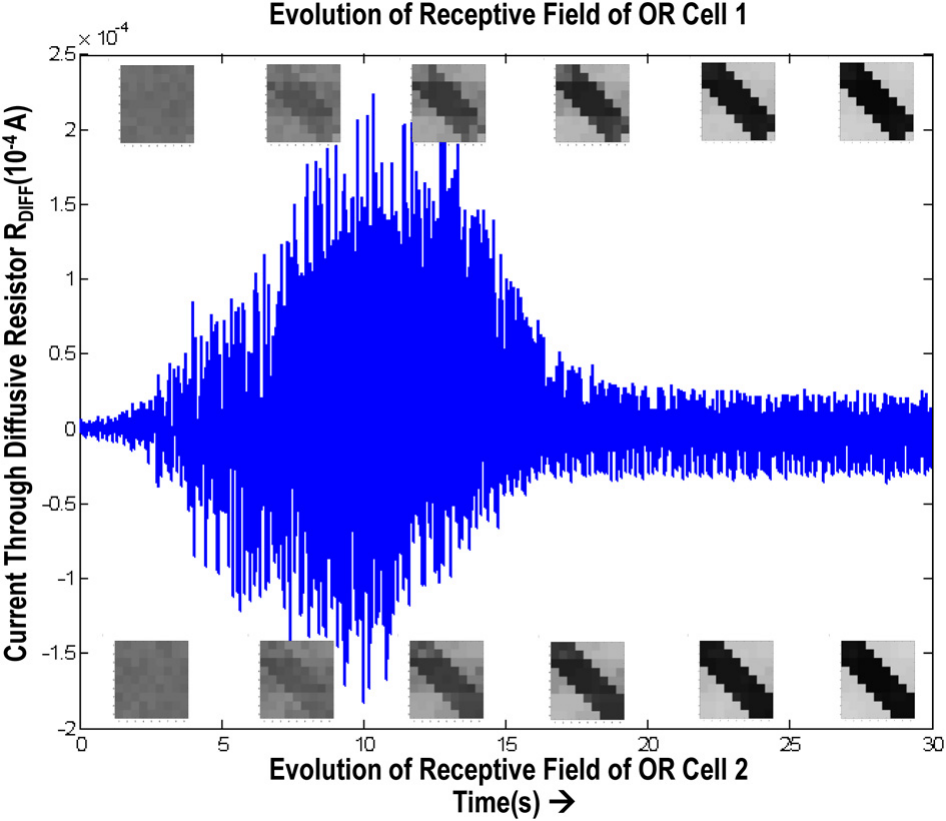
**Shows the variation of current in the resistance, R_diff_, connecting two orientation selective cells**. The top boxes show the evolution of receptive field of orientation cell 1 and the bottom boxes show the evolution of receptive field of orientation cell 2. The current is high (~150 μA) during the learning phase. Once the orientation has been learnt, or the floating gate voltages have matured, the current reduces and remains constant thereafter.

In order to test if our orientation cell fulfills the premise laid down for diffusive interaction between cells, we performed multiple simulations with orientation cells having different initial biases but similar random inside epoch order of inputs, and we let them develop under two conditions, (1) independently, i.e., without any diffusive interaction and, (2) with diffusive interaction. As discussed previously, the voltage at node dno affects the feedback that regulates the response of the cell. If we connect two orientation cells at the diffusive node (dno) by means of a resistance, then on receiving similar inputs, the cell with the higher voltage at node dno, starts to influence the response of the other cell by making the injection and tunnel feedback mechanisms of both the cells similar, thus enforcing the same pattern on each of the cells. By changing the value of the diffusion resistance (by increasing the resistance we reduce diffusion constant and by reducing its value we increase the diffusion) we can modify the extent of interaction we want between the cells. Several experiments were performed with different diffusion constants, different biases and different inputs. Each time for moderate(300> *R*_diff_ >100 Ohms) and high values of diffusion constant(100 > *R*_diff_ >0 Ohms), it was found that the response of the two cells became similar. To which side the orientation preference tilts is dependent on which cell has a stronger bias. The simulations were done for two and three cells connected in a row. Figure 12 shows some of the interesting results. Irrespective of the way the cells develop independently, whether one is ON-Centered and other OFF-Centered, whether their orientation preferences are totally opposite of each other i.e., 135° and 45°, with diffusion, they become selective to the same orientation. It is important to note that the lateral diffusive network and the feedback network are only important as long as the learning is taking place and the receptive field of the cells are developing. Once the receptive fields have evolved, the lateral connectivity i.e., RC diffusive network and the cell's feedback network become ineffective and the cell work's in a feed forward mode where in on applying a set of inputs, the cell responds according to its developed orientation preference. The power dissipation also varies according to the learning profile of the cell e.g., between two orientation cells connected by a 100 ohm resistance, the current through the diffusive resistor is maximum (~150 μA) during learning but reduces drastically (~10 μA) once the learning is over (Figure 13). The power can be reduced by shifting the whole resistance regime of the cell to larger values but keeping the necessary ratio between (τ_Diffusion_) and (τ_Reaction_) intact.

## 6. Results and discussion

Time-staggered or uncorrelated inputs have been shown to be essential for feature map formation (Stryker and Strickland, 1984; Weliky and Katz, 1997; Buffeli et al., 2002; Zhang et al., 2002). The time-staggered Winner Takes All algorithm, based on un-correlated inputs, has previously been shown to be biologically more realistic and a mechanism underlying formation of Ocular Dominance Maps (Markan et al., 2013). This paper introduces the design of a cortical cell that is built using ts-WTA cells comprising of ON/OFF Centered synapses forming a three layered structure similar to the visual sensory system in the brain. On application of patterns resembling different orientations, the floating gate dynamics, the diffusive interaction and the feedback regime act in a way that the cell is able develop orientation selectivity. Repeated simulations show that the orientation selectivity develops according to two major factors, initial biases(nature) and the inputs applied(nurture) and that there is an equal likelihood of the circuit becoming selective to any of the eight patterns applied. Embedded in a RC grid, these orientation selective cells are able to modify their feature preference under strong neighborhood influence to form clusters of cells with similar feature preference. The cell also responds to periodic patterns and spatial frequency just like experimentally observed cells of the visual cortex. This is a significant step toward developing neuromorphic equivalents of biological phenomenon that could have diverse applications in artificial vision systems.

Diffusive hebbian learning based on reaction-diffusion and competition for neurotropic factors (Markan, 1996; Markan and Bhaumik, 1999; Bhaumik and Mathur, 2003), has strong biological support as basis to explain local computation and organization in the brain. It is now well known that the developing cortex is a generic neural structure that gets compartmentalized for processing different sensory inputs through an adaptive learning process. It therefore becomes important to explore the basic learning paradigms that are active in the brain, which are able to extract statistically relevant information from the sensory input space and map it onto the cortex, so that such principles can be applied in artificial systems. In this sense, the model developed is very generic and can be applied to inputs from any sensory modality such as olfaction, gustatory, somatosensory and auditory. Some preliminary work also demonstrates the applicability of the model to abstract pattern recognition. In the brain no sensory system works in isolation. Rather, it is a combination of sensory inputs to different sensory modalities that the brain responds best to. Eventual integration of features maps, corresponding to different sensory systems, onto a common platform could act as a database for higher cognitive algorithms to work on. The work presented in this paper is a small yet significant step toward the goal of building truly cognitive neuromorphic systems because it presents a novel approach towards incorporating adaptability and learning in artificial systems by modeling the developmental aspects of feature selectivity and feature map formation in the brain. While reaction diffusion has been able to address local range, non-axonal interactions in the brain and explain how cortical feature maps evolve to a large extent, more recent research has highlighted the role of gap junctions in lateral information processing in the brain (Hameroff, 2010; Ebner and Hameroff, 2011; Gupta and Markan, 2013). Experiments have revealed that sibling neurons connected by gap junctions develop to have the same feature preference (Li et al., 2012; Mrsic-Flogel and Bonhoeffer, 2012). Since gap junctions can form networks of neurons spanning large areas of the cortex, understanding how they function, could give us new insights into multi-modal information processing in the brain. It seems interesting to explore gap junctions and see how similar behavior can be emulated in hardware.

### Conflict of interest statement

The authors declare that the research was conducted in the absence of any commercial or financial relationships that could be construed as a potential conflict of interest.

## Supplementary material

A video showing the development of oriention receptive field has been made available as a part of the online supplementary data. A document on the Monte-Carlo Analysis of the cell under device parameter variations has also been provided in the supplementary section. The Supplementary Material for this article can be found online at: http://www.frontiersin.org/journal/10.3389/fnins.2014.00054/abstract
