# Supplementary material for: An adaptable neuromorphic model of orientation selectivity based on floating gate dynamics
Source: Front Neurosci. 2014 Apr 2;8:54. doi: 10.3389/fnins.2014.00054 (PMC3980111; doi:10.3389/fnins.2014.00054)
Supplement: Supplementary file 1 [file Presentation1.ZIP › Gupta_Monte-Carlo Analysis.DOCX]

**Monte Carlo Analysis of Orientation Selective Cell**

To test the robustness of our Orientation Selective Cell, Monte Carlo Analysis with random parameter value variations was performed. The Orientation Selective Cell’s performance heavily depends on the injection and tunnel currents. Any variation in these currents can affect the equilibrium of the circuit and affect the circuit’s learning and response behavior. Our models for injection and tunnel currents are based on the equations described in [Rahimi et.al, 2001] also described below.

The tunnel current varies according to the below equation and depends on the floating gate voltage, the tunnel voltage and a factor V_f_ that depends on the oxide thickness. Our model assumes an oxide thickness of 70A^o^. *I_to_* is a pre-exponential current. The typical values of these parameters are listed in table I.

(1)

The injection current varies according to the below equation. It depends on the gate (floating)to drain and source to drain voltages. Here η, β and δ are fit parameters and =1 for units consistency. I_S_ is the source current which is ~10nA and can be ignored for all practical purposes. The typical values of the parameters η, β and δ are listed in table I.


 (2)

Table I. Base values of all the device parameters

| **Parameter** | **I_to_** | **V_f_** | **η** | **β** | **δ** |
| --- | --- | --- | --- | --- | --- |
| **Base Value** | 9.35x10^8^ | 368.04 | 1.30x 10^-5^ | 155.75 | 0.702 |

A single Orientation Cell is made from 9x9 ts-WTA cells. During fabrication, the variation in parameters can happen in two ways.

i). There could be variation in the parameter base values over the whole IC

ii). There could be minor variations in parameter values across the same IC

To test the robustness of our design under these two situations we performed Monte-Carlo analysis at two levels. First, by randomly varying the base values of all the parameters and applying the same (randomly generated parameters ) to all the 9x9 ts-WTA cells and second, by randomly varying the base values and applying different parameter values to all 9x9 ts-WTA cells. The first analysis determines the extent to which the orientation cell is resilient to changes in the base values of parameters and the second analysis checks for how resilient the circuit is to variations in parameters across the 9x9 ts-WTA cells over the same IC. The following sections describe the detailed analysis.

**1. Performance under parameter base value variation**

To check for the first case, a MATLAB code was written to generate random values of all the parameters. The parameters were varied by 10%, 5% and 3% from the base values listed in table I. Some sample values of the parameters are listed in tables 2a, 2c and 2e.

**1.1** **Performance under 10% parameter variation**

Multiple simulations were performed on the Orientation cell keeping the inputs and initial conditions the same but varying the parameters and applying the same to all the 9x9 ts-WTA cells.

**Table 2a.** 10% variation in device parameters

|  | **I_to_** | **V_f_** | **η** | **β** | **δ** |
| --- | --- | --- | --- | --- | --- |
| 1 | 987114888.228762 | 362.893919 | 0.000013 | 145.053742 | 0.677823 |
| 2 | 875529355.768128 | 364.844702 | 0.000014 | 140.724017 | 0.663066 |
| ***3*** | ***925538930.197264*** | ***343.556415*** | ***0.000012*** | ***166.233249*** | ***0.736087*** |
| ***4*** | ***947332216.259480*** | ***396.207331*** | ***0.000014*** | ***144.205576*** | ***0.730049*** |
| ***5*** | ***1016157953.038652*** | ***385.248023*** | ***0.000013*** | ***161.530224*** | ***0.663095*** |
| ***6*** | ***844396973.116741*** | ***358.564317*** | ***0.000014*** | ***169.719006*** | ***0.699921*** |
| ***7*** | ***849825326.820521*** | ***350.038661*** | ***0.000012*** | ***159.342702*** | ***0.764429*** |
| 8 | 882827929.914150 | 362.929978 | 0.000014 | 148.026625 | 0.680760 |
| ***9*** | ***966722055.874403*** | ***374.177837*** | ***0.000014*** | ***145.640335*** | ***0.747585*** |
| ***10*** | ***856079594.191899*** | ***358.490477*** | ***0.000012*** | ***163.461846*** | ***0.692258*** |

**Analysis:** The cell’s learning response remained fairly stable for cases 1, 2 and 8 however for rest of the cases the response was significantly altered. Prior analysis done on a single ts-WTA cell reported in [Markan, C.M., Gupta, P., Bansal, M., 2013] shows that ts-WTA is stable under 10% variations in all parameters except the value of parameter V_f._ However, as can be seen from equation(1), even when V_f_ changes, the overall effect of the exponential term in the tunnel current can be kept constant by changing the tunnel voltage(V_tun_) appropriately. By modifying V_tun_, for all the cases except for case 4, the response of the cell could be made normal. Therefore, it seems that the circuit is not very stable to 10% variation in V_f._ However, in 90% of the cases, we can recover from this unstable response by adjusting V_tun._  The exact variation in V_tun_ can be seen in table 2b.

**Table 2b.** Change in the value of V_tun_ from the original value of 13.6v

| ***Case No*** | ***3*** | ***5*** | ***6*** | ***7*** | ***9*** | ***10*** |
| --- | --- | --- | --- | --- | --- | --- |
| ***Modified V_tun_(volts)*** | 13 | 14 | 13.3 | 13.3 | 13.8 | 13.3 |
| $\boldsymbol{\delta}$***V_tun_(volts)*** | -0.6 | 0.4 | -0.3 | -0.3 | 0.2 | -0.3 |

**1.2 Performance under 5% parameter variation**

To check the if the performance of the orientation cell improves with a lower percentage parameter variation, we varied the parameters by 5%. Some of the sample parameter values used in the simulations are recorded in table 2c.

**Table 2c.** 5% variation in device parameters

|  | **I_to_** | **V_f_** | **η** | **β** | **δ** |
| --- | --- | --- | --- | --- | --- |
| ***1*** | ***903448852.215753*** | ***351.649108*** | ***0.000013*** | ***155.776379*** | ***0.734687*** |
| ***2*** | ***945800047.304039*** | ***380.988332*** | ***0.000013*** | ***160.131355*** | ***0.696717*** |
| ***3*** | ***888654705.346983*** | ***377.145651*** | ***0.000013*** | ***158.178012*** | ***0.706970*** |
| 4 | 953635366.477950 | 360.011766 | 0.000013 | 158.136659 | 0.733318 |
| 5 | 911184212.488790 | 368.653780 | 0.000013 | 148.067916 | 0.719875 |
| 6 | 924005782.434137 | 358.665951 | 0.000013 | 154.696435 | 0.708818 |
| 7 | 912696144.559396 | 360.404296 | 0.000013 | 148.440872 | 0.681787 |
| ***8*** | ***945049412.311427*** | ***383.577475*** | ***0.000013*** | ***149.460724*** | ***0.718531*** |
| 9 | 945380010.191412 | 362.462831 | 0.000013 | 152.474247 | 0.711202 |
| 10 | 914740138.349817 | 375.717765 | 0.000013 | 159.974569 | 0.683564 |

**Analysis:** The cells response remained fairly stable for 60% of the cases, however, in 40% cases (e.g. case 1, 2, 3 and 8) the learning was altered moderately. In these cases also the response could be corrected by modifying V_tun_ appropriately. The exact change in V_tun_ is listed in table 2d.

**Table 2d.** Change in the value of V_tun_ from the original value of 13.6v

| ***Case No*** | ***1*** | ***2*** | ***3*** | ***8*** |
| --- | --- | --- | --- | --- |
| ***Modified V_tun_(volts)*** | 13.3 | 13.8 | 13.8 | 14 |
| $\boldsymbol{\delta}$***V_tun_(volts)*** | -0.3 | 0.2 | 0.2 | 0.6 |

**1.3 Performance under 3% parameter variation**

To find the range of parameter variation within which the cell works perfectly (without having to change V_tun_)we then varied the parameters by 3%. Some sample values are listed in table 2e.

**Table 2e.** 3% variation in device parameters

|  | **I_to_** | **V_f_** | **η** | **β** | **δ** |
| --- | --- | --- | --- | --- | --- |
| 1 | 942305113.731676 | 357.863394 | 0.000013 | 155.912888 | 0.700073 |
| 2 | 919794968.390765 | 361.952084 | 0.000013 | 154.635215 | 0.704436 |
| 3 | 956106758.514632 | 376.302343 | 0.000013 | 151.379141 | 0.702056 |
| 4 | 917102887.966741 | 372.542313 | 0.000013 | 151.709425 | 0.702733 |
| 5 | 912307660.991632 | 359.245728 | 0.000013 | 154.072777 | 0.681407 |
| 7 | 938824854.542024 | 365.572080 | 0.000013 | 155.638304 | 0.706797 |
| 8 | 947710289.185848 | 365.296566 | 0.000013 | 157.926790 | 0.700346 |
| 9 | 933122974.181908 | 360.571770 | 0.000013 | 153.492817 | 0.721009 |
| 10 | 943420647.202232 | 362.339327 | 0.000013 | 155.784875 | 0.690321 |

**Analysis:** It was found that in all the cases the cell’s behavior was as expected. For case 3, the learning took slightly longer, however the output or receptive field did converge to the expected pattern. For all other cases the learning was normal.

**2. Performance under parameter variation across the same IC**

To test how robust our circuit is to parameter variations between the different 9x9 ts-WTA cells a MATLAB code was written to generate random parameters for all the 81 ts-WTAs. The parameter variation range was varied from $\pm$2% to $\pm$10%. It was found that when the parameters varied within $\pm$3% of the base values, the cell performed normally, however, for larger limits the cell’s performance deteriorated. Sample values with a $\pm$3% random parameter variation across all 9x9 ts-WTA cells are listed in table 3.

**Table 3.** Sample values of Monte-Carlo analysis with $\pm$3%

parameter variation over all 81 ts-WTA cells together

| tsWTA | **I_to_** | **V_f_** | **η** | **β** | **Δ** |
| --- | --- | --- | --- | --- | --- |
| **1** | 916926258.638770 | 374.388765 | 0.000013 | 154.220189 | 0.686514 |
| **2** | 938515684.395454 | 373.868665 | 0.000013 | 158.703084 | 0.707317 |
| **3** | 943198321.797023 | 371.029368 | 0.000013 | 153.853756 | 0.685235 |
| **4** | 936013995.315444 | 363.245351 | 0.000013 | 152.389534 | 0.710212 |
| **5** | 943216764.482654 | 360.545627 | 0.000013 | 151.369412 | 0.681143 |
| **6** | 934960249.771276 | 370.054539 | 0.000013 | 152.911303 | 0.685080 |
| **7** | 942163018.938486 | 367.354815 | 0.000013 | 151.891182 | 0.698130 |
| **8** | 934978692.456907 | 359.570799 | 0.000013 | 159.771959 | 0.680987 |
| **9** | 939661329.858476 | 378.813901 | 0.000013 | 154.922632 | 0.701025 |
| **10** | 961250755.615160 | 378.293801 | 0.000013 | 159.405526 | 0.721829 |
| **11** | 909833393.016729 | 375.454503 | 0.000013 | 154.556199 | 0.699747 |
| **12** | 958749066.535150 | 367.670486 | 0.000013 | 153.091976 | 0.682604 |
| **13** | 909851835.702361 | 364.970763 | 0.000013 | 152.071855 | 0.695654 |
| **14** | 957695320.990982 | 374.479675 | 0.000013 | 153.613746 | 0.699592 |
| **15** | 908798090.158193 | 371.779951 | 0.000013 | 152.593624 | 0.712642 |
| **16** | 957713763.676613 | 363.995934 | 0.000013 | 151.129402 | 0.695499 |
| **17** | 962396401.078182 | 361.156637 | 0.000013 | 155.625075 | 0.715537 |
| **18** | 927885826.834867 | 360.636536 | 0.000013 | 160.107969 | 0.694220 |
| **19** | 932568464.236436 | 357.797239 | 0.000013 | 155.258642 | 0.714258 |
| **20** | 925384137.754857 | 372.095622 | 0.000013 | 153.794419 | 0.697116 |
| **21** | 932586906.922067 | 369.395898 | 0.000013 | 152.774298 | 0.710166 |
| **22** | 924330392.210689 | 378.904810 | 0.000013 | 154.316189 | 0.714103 |
| **23** | 931533161.377899 | 376.205087 | 0.000013 | 153.296067 | 0.685033 |
| **24** | 924348834.896320 | 368.421070 | 0.000013 | 151.831845 | 0.710011 |
| **25** | 929031472.297889 | 365.581773 | 0.000013 | 156.327517 | 0.687929 |
| **26** | 950620898.054573 | 365.061672 | 0.000013 | 151.465412 | 0.708732 |
| **27** | 955303535.456142 | 362.222375 | 0.000013 | 155.961084 | 0.686650 |
| **28** | 948119208.974563 | 376.520758 | 0.000013 | 154.496862 | 0.711627 |
| **29** | 913608634.731247 | 376.000657 | 0.000013 | 158.979756 | 0.690310 |
| **30** | 947065463.430395 | 361.247546 | 0.000013 | 155.018631 | 0.686495 |
| **31** | 939881136.948816 | 375.545929 | 0.000013 | 153.554409 | 0.711472 |
| **32** | 947083906.116026 | 372.846206 | 0.000013 | 152.534287 | 0.682402 |
| **33** | 910053200.107069 | 372.186531 | 0.000013 | 153.187976 | 0.710193 |
| **34** | 917255969.274280 | 369.486808 | 0.000013 | 152.167854 | 0.681124 |
| **35** | 907551511.027059 | 361.563217 | 0.000013 | 156.219426 | 0.713089 |
| **36** | 914754280.194270 | 358.863493 | 0.000013 | 155.199304 | 0.684019 |
| **37** | 936343705.950954 | 358.343393 | 0.000013 | 159.682199 | 0.704822 |
| **38** | 952147850.323374 | 375.212011 | 0.000013 | 156.885460 | 0.707324 |
| **39** | 945094128.509911 | 370.251336 | 0.000013 | 157.778615 | 0.721689 |
| **40** | 946588241.534838 | 361.889786 | 0.000013 | 151.110136 | 0.710229 |
| **41** | 948082793.619080 | 378.515306 | 0.000013 | 158.072742 | 0.721210 |
| **42** | 926641976.156827 | 368.470338 | 0.000013 | 158.521796 | 0.705382 |
| **43** | 956910280.479333 | 370.277375 | 0.000013 | 152.741519 | 0.712188 |
| **44** | 921082367.368290 | 377.230513 | 0.000013 | 152.746472 | 0.708287 |
| **45** | 951350671.690796 | 379.037550 | 0.000013 | 156.311195 | 0.715094 |
| **46** | 938458128.126249 | 368.496377 | 0.000013 | 153.484701 | 0.695881 |
| **47** | 931404406.312785 | 363.535702 | 0.000013 | 154.377856 | 0.710246 |
| **48** | 932898519.337712 | 377.256552 | 0.000013 | 157.054377 | 0.698786 |
| **49** | 961718880.124376 | 361.631085 | 0.000013 | 153.783781 | 0.691501 |
| **50** | 912952253.959702 | 361.754704 | 0.000013 | 155.121037 | 0.693939 |
| **51** | 914446366.984628 | 375.475554 | 0.000013 | 157.797558 | 0.682479 |
| **52** | 907392645.171165 | 370.514879 | 0.000013 | 158.690713 | 0.696844 |
| **53** | 908886758.196092 | 362.153329 | 0.000013 | 152.022234 | 0.685385 |
| **54** | 952094214.631544 | 373.694556 | 0.000013 | 158.540739 | 0.708292 |
| **55** | 945040492.818081 | 368.733881 | 0.000013 | 159.433894 | 0.722657 |
| **56** | 946534605.843008 | 360.372331 | 0.000013 | 152.765415 | 0.711198 |
| **57** | 919254966.629671 | 366.829264 | 0.000013 | 158.839819 | 0.703912 |
| **58** | 926588340.464997 | 366.952883 | 0.000013 | 160.177075 | 0.706350 |
| **59** | 928082453.489924 | 358.591333 | 0.000013 | 153.508596 | 0.694890 |
| **60** | 921028731.676461 | 375.713058 | 0.000013 | 154.401751 | 0.709255 |
| **61** | 922522844.701387 | 367.351508 | 0.000013 | 157.078272 | 0.697796 |
| **62** | 951343205.488051 | 373.808442 | 0.000013 | 153.807676 | 0.690510 |
| **63** | 958676579.323377 | 373.932060 | 0.000013 | 155.144933 | 0.692948 |
| **64** | 960170692.348304 | 365.570510 | 0.000013 | 157.821454 | 0.681489 |
| **65** | 932891053.134967 | 372.027444 | 0.000013 | 154.550858 | 0.716323 |
| **66** | 940224426.970293 | 372.151062 | 0.000013 | 155.888114 | 0.718761 |
| **67** | 941718539.995220 | 363.789512 | 0.000013 | 158.564635 | 0.707301 |
| **68** | 934664818.181756 | 358.828837 | 0.000013 | 159.457790 | 0.721667 |
| **69** | 936158931.206683 | 372.549687 | 0.000013 | 152.789311 | 0.710207 |
| **70** | 908879291.993347 | 379.006621 | 0.000013 | 158.863715 | 0.702921 |
| **71** | 916212665.828673 | 357.047839 | 0.000013 | 160.200971 | 0.705359 |
| **72** | 917706778.853599 | 370.768689 | 0.000013 | 153.532492 | 0.693900 |
| **73** | 919201330.937842 | 365.311809 | 0.000013 | 151.150098 | 0.704880 |
| **74** | 912147170.065063 | 357.446464 | 0.000013 | 157.102168 | 0.696805 |
| **75** | 928028817.798094 | 357.073877 | 0.000013 | 155.163876 | 0.695859 |
| **76** | 948300904.687052 | 364.027016 | 0.000013 | 155.168828 | 0.691958 |
| **77** | 922469209.009558 | 365.834052 | 0.000013 | 158.733552 | 0.698764 |
| **78** | 922320701.160361 | 377.063633 | 0.000013 | 156.628128 | 0.715260 |
| **79** | 942484766.291424 | 376.017635 | 0.000013 | 159.875657 | 0.702075 |
| **80** | 929585636.105684 | 366.066372 | 0.000013 | 156.706191 | 0.689867 |
| **81** | 938236223.258724 | 357.886154 | 0.000013 | 158.417267 | 0.705530 |

**3. Conclusions**

The two stage Monte-Carlo analysis performed on the Orientation Selective Cell brings forth the following conclusions

i). The cell is fairly stable under a $\pm3\%$ variation in parameter base values.

ii). For a variation greater than 3% but less than 10%, the response of the cell gets
 altered but it can easily be recovered by changing the tunnel voltage V_tun_ appropriately.

iii). The cell is also resilient upto a $\pm3\%$ parameter mismatch between the 9x9 ts-
 WTA cells forming the orientation cell.

**4. References**

[1]. Rahimi, K., Diorio, C., Hernandez, C., & Brockhausen, M. D. (2002). A simulation model for
 floating-gate MOS synapse transistors. In *Circuits and Systems, 2002. ISCAS 2002. IEEE
 International Symposium on* (Vol. 2, pp. II-532). IEEE.

[2]. Markan, C. M., Gupta, P., & Bansal, M. (2013). An adaptive neuromorphic model of Ocular
 Dominance map using floating gate ‘synapse’. *Neural Networks*.
